# Stereoselective Synthesis of Selenium-Containing Glycoconjugates via the Mitsunobu Reaction

**DOI:** 10.3390/molecules26092541

**Published:** 2021-04-27

**Authors:** Luigia Serpico, Mauro De Nisco, Flavio Cermola, Michele Manfra, Silvana Pedatella

**Affiliations:** 1 Department of Chemical Sciences, University of Napoli Federico II, Via Cintia 4, I-80126 Napoli, Italy; luigia.serpico@unina.it (L.S.); cermola@unina.it (F.C.); pedatell@unina.it (S.P.); 2 Department of Sciences, University of Basilicata, Via dell’Ateneo Lucano 10, I-85100 Potenza, Italy; michele.manfra@unibas.it

**Keywords:** organoselenium compounds, selenosugar, mitsunobu reaction, antioxidants

## Abstract

A simple and efficient route for the synthesis of new glycoconjugates has been developed. The approach acts as a model for a mini-library of compounds with a deoxy-selenosugar core joined to a polyphenolic moiety with well-known antioxidant properties. An unexpected stereocontrol detected in the Mitsunobu key reaction led to the most attractive product showing a natural d-configuration. Thus, we were able to obtain the target molecules from the commercially available d-ribose via a shorter and convenient sequence of reactions.

## 1. Introduction

In the last decade, a large amount of evidence has suggested a link to oxidative stress (OS) and ageing-related neurodegenerative diseases (NDs) [1,2], which are characterized by progressive damage in neural cells as well as by a selective loss of neuronal populations. Since OS refers to an imbalance between the production and removal of reactive oxygen species (ROS), antioxidant therapies have been proposed to prevent or attenuate their induced damage [3,4].

**Figure 1:**
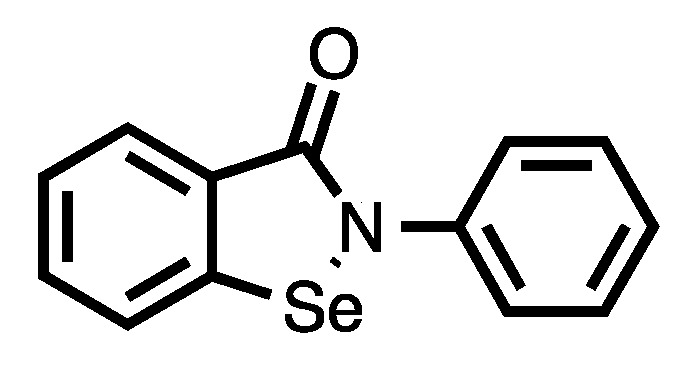
Ebselen.

Recently, the therapeutic effects of several small molecules, normally taken with the diet, have been studied and their potential applications proposed. In more detail, several selenium-containing compounds have been synthesized, and they turned out to be potent neuroprotective agents with a modest effect on normal tissues and are clinically well tolerated [5,6]. Among them, the best known is the Ebselen [7] (Figure 1), a small organoselenium compound that acts as a glutathione peroxidase (GPx) mimetic. Likewise, several polyphenolic molecules have shown a strong antioxidant activity [8,9], including caffeic acid, which has the potential to modulate oxidation and inflammation, as demonstrated in several studies [10]; curcumin, with proven anticancer and anti-inflammatory characteristics [11,12]; and dopamine, whose loss is involved in the early phase of Alzheimer’s Disease (AD) development [13].

**Scheme 1:**
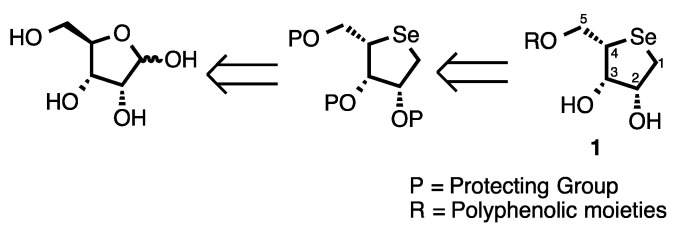
Retrosynthesis of target.

Faced with setting up a library of new antioxidant glycoconjugates to address the formulation of supplements focused on neurodegenerative disorders such as AD, we investigated the design and synthesis of new molecules combining the antioxidant properties of selenium with the chelating and antioxidant properties of a phenolic structure moiety, as displayed in the Scheme 1.

The introduction of selenium into organic structures has been performed via chemo-, regio-, and stereoselective methods, such as nucleophile, electrophile, and radical techniques only during the past three decades, challenging the well-known instability and unusual behavior of early derivatives [14]. The results of Matsuda [15], Pinto [16], and Jeong [17] represent the starting approach towards the chemistry of selenium-ribose compounds.

Inspired by these studies and taking into account that, to the best of our knowledge, no examples of 4-selenosugar-5-conjugates have been described in the literature to date; we wish to report their first synthesis here.

## 2. Results and Discussion

As outlined in the retrosynthetic path (Scheme 1), we started from the commercially available d-ribose. In fact, the latter was converted into the corresponding deoxy-selenosugar derivative **1**, modifying a procedure reported by Jeong et al. [18].

**Scheme 2:**
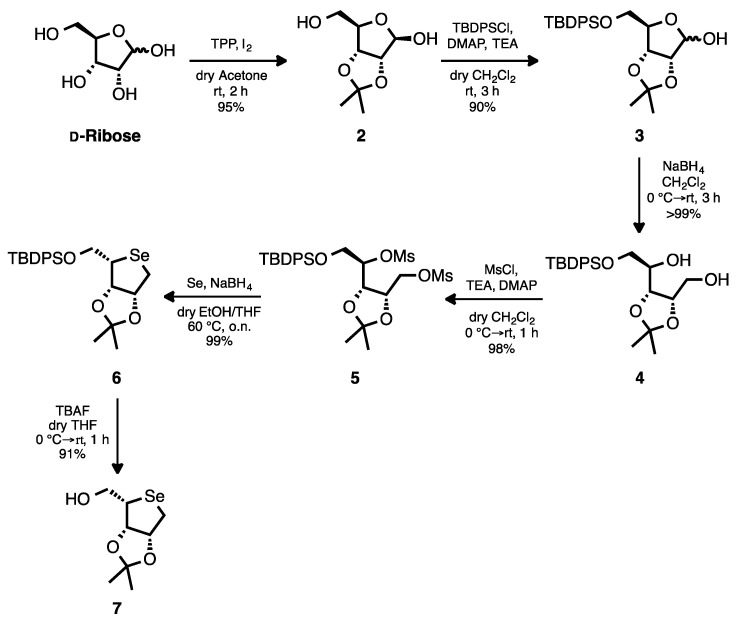
Synthesis of building block 7.

Firstly, we protected d-ribose with orthogonal groups, with a view of selectively removing them during the synthetic strategy. In detail, isopropylidene was used to protect hydroxyl groups in C-2 and C-3 through a protocol [19] already described, characterized by smooth reaction conditions with a low environmental impact leading to compound **2** with a 95% yield. The *O*-isopropilydene derivative, without any further time consuming work up or purification procedures and after treatment with *tert*-butyl(chloro)diphenylsilane (TBDPSCl), triethylamine (TEA), and dimethylaminopyridine (DMAP) in anhydrous CH_2_Cl_2_, afforded the silyl ether **3**, which in turn provided the diol **4** with an excellent yield (>99%) under reductive conditions (sodium borohydride). The next step where the diol 4 was converted to the corresponding dimesylate **5** allowed us to enter good leaving groups on C-1 and C-4 so that the subsequent treatment of **5** with selenium in the presence of sodium borohydride in EtOH-THF at 60 °C gave the deoxy-selenosugar derivative **6** at a 95% yield. It is noteworthy that the reaction takes place with inversion of the configuration [16,17,18], thus leading to the corresponding l-deoxy-selenosugar. Using dry conditions allowed the optimization of the procedure [18], providing **6** with a 99% yield (Scheme 2). Finally, the removal of the TBDPS protecting group on C-5 exploiting the great affinity of fluoride to silicon afforded the seleno building block 7 with a high yield (75% overall yield).

To perform the reaction giving the glycoconjugate, we attempted to convert **7** to an iododerivative using a triphenylphosphine polymer-bound/iodine complex already reported on different substrates [20], but the only product found was the dimer, probably resulting from the quick formation of the corresponding iodide that was attacked by the OH group of the remaining **7**. It was not possible to evaluate the efficiency of mesyl or tosyl derivatives, since both were prepared in very low yields. The failed results prompted us to take a different approach based on the Mitsunobu reaction, which is well-known and carried out with an activated alcohol prepared in situ using triphenylphosphine (TPP) and an azodicarboxylate as promoter.

**Scheme 3:**
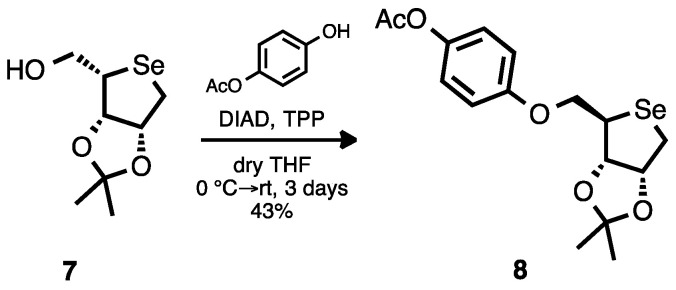
Mitsunobu reaction.

Therefore, our investigation began with the coupling of the seleno derivative **7** TPP-complex with monoacetylated hydroquinone [21] chosen as a model compound. The reaction in dry THF after three days gave a 43%-yield compound **8** as a major and unexpected product (Scheme 3).

**Scheme 4:**
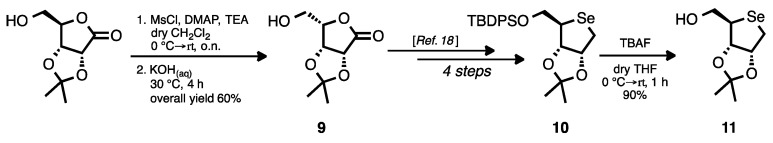
Synthesis of the isomer of 7.

NMR was used to assign the configuration at C4 in the latter. It is well-known that the stereostructure of the furanose moiety is often difficult to analyze due to the low energy barriers between various puckering states [22]. It has already been reported in the literature that the bulky selenium atom in the 4′-selenoribonuclesosides led to a south (C2′-endo) pucker state [17]. However, since significant NOE effects were not detected for the hydrogens at C2 and C4 and the coupling constants of **8** were not supportive for the stereochemistry assignment with certainty, we decided to evaluate the outcome of the d-deoxy-selenosugar derivative **11** coming from the commercially available d-ribonolactone (Scheme 4). Thus, we employed the procedure reported by Batra et al. [23] to accomplish a first inversion of the configuration leading to the intermediate **9**, which, in turn, was converted with a high yield into the corresponding d-target **11**, using the same route previously described for his isomer **7**.

A comparative analysis of ^1^H NMR coupling constants in compounds **6** and **10** as well as **7** and **11** (Table 1) was therefore carried out to investigate the range of the vicinal coupling constants. The ^3^**J*_3,2_* medium value was 5.6 Hz, the ^3^**J*_2,1b_* was 4.8 Hz, and the ^3^**J*_2,1a_* was 2.0 Hz. More interestingly, however, was that for **6** and **7**, the ^3^**J*_3,4_* medium value was 4.3 Hz, while for **10** and **11**, the same coupling value was 1.6 Hz. Taking this into account, in glycoconjugates, the ^3^**J*_3,4_* medium value of 1.6 Hz is consistent with the 3,4-*anti*-configuration [15].

The selectivity seen in the model reaction, whose mechanism is under investigation, was also detected when the scope of our approach was explored. Indeed, all polyphenolic substrates used gave the sole corresponding d-deoxy-seleno glycoconjugate (**8**, and **12**–**15**, Table 2). Moreover, when we performed the Mitsunobu reaction on the same polyphenols using the isomer **11** as the starting seleno substrate, the spectral and analytical data clearly indicated we had obtained the same product **8** and **12**–**15** with corresponding comparable yields (Table 2). These results, presumably resulting from the already reported unusual behavior of selenium [24], support the use in future of the “l-route” (seven steps up to isomer **7**, overall yield 75%) to obtain further products, thereby avoiding the alternative time and products consuming “d-route” (eight steps up to isomer **11**, overall yield 47%).

In the preliminary attempts performed on hydroquinone used as a model compound, the presence of a hydroxyl group different from the one involved in the reaction appeared to be a limit of reactivity for the methodology, thus affording a very low yield of the desired conjugated. On the other hand, the reaction on the monoacetylated hydroquinone proceeded with fair yields.

A different approach was reserved for curcumin, whose hydroxyl groups in C-4 have not been intentionally protected due to known handling problems of the molecule. Nevertheless, the yield was reasonable with the challenging purification procedure currently under improvement.

Concerning the polyphenols having the COOH group in addition to the phenolic one (caffeic and ferulic acids), as expected, due to the lower pka of the carboxyl group, they proved to be excellent substrates for the reaction (yields 67–72%), taking into account that we used a protecting-free approach, which is very useful in multistep programs.

**Scheme 5:**
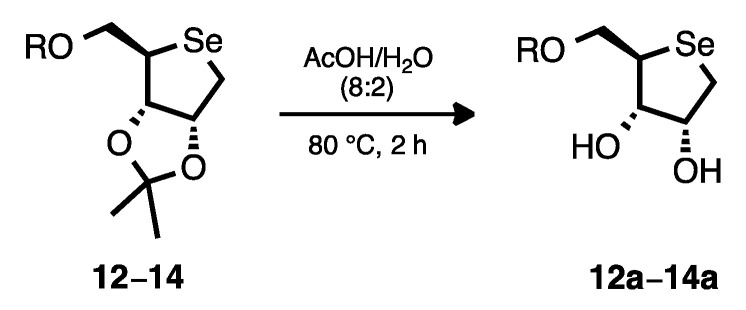
Removal of protecting group.

The removal of the isopropylidene group on C-2 and C-3 (Scheme 5) under acid conditions provided, except for the curcumin, the corresponding derivatives **12a**–**14a** (yield 68–80%) that will be investigated for their reducing and scavenging abilities consistent with the mechanism generally accepted for antioxidant compounds.

## 3. Materials and Methods

All the reagents were acquired (Aldrich, Fluka, Sigma) at the highest purity available and used without further purification. Thin-layer chromatography was performed with silicagel plates Merck 60 F_254_, and the display of the products on TLC was accomplished with UV lamp lighting, molecular iodine, and in H_2_SO_4_–EtOH (95:5) with further heating until the development of color. The column chromatographies were carried out using 70–230 mesh silica gel (Merck). The NMR spectra (Supplementary Materials) were recorded on Bruker DRX (400MHz) and Varian Inova Marker (500MHz) spectrometers in CDCl_3_ solution unless otherwise specified. The chemical shifts are reported in ppm (δ). The ^1^H and ^13^C NMR full characterization of the products was obtained based on 2D NMR and 1D selective NOESY. The TopSpin 4.0.6 software package was used for the analysis.

*Preparation of* 2,3*-O-isopropylidene-b-d-ribofuranose* (**2**): To a magnetically stirred suspension of anhydrous triphenylphosphine (0.525 g, ca. 2.0 mmol) in anhydrous acetone (6.0 mL) at r.t., a solution of I_2_ (0.508 g, 2.0 mmol) in the same solvent (18.0 mL) was added dropwise in the dark and under dry N_2_ atmosphere. After 15 min, solid d-ribose (0.150 g, 1.0 mmol) was added in one portion to the suspension. TLC monitoring (CHCl_3_–MeOH, 9:1) showed that the starting sugar was completely consumed within 30 min. The reaction mixture was then filtered, washed with acetone, and dried (Na_2_SO_4_). The evaporation of the solvent under reduced pressure gave a crude residue that was used directly in the next step without further purification (0.181 g, 0.95 mmol, 95%). ^1^H NMR (500 MHz): *δ* 1.33 (s, 3 H, CH_3_), 1.48 (s, 3 H, CH_3_), 3.70 (dd, *J*_5a,5b_ = 11.6, *J*_5a,4_ = 1.5, 1H, H5a), 3.79 (dd, *J*_5b,5a_ = 11.6, *J*_5b,4_ = 1.8, 1 H, H5b), 4.30 (m, 1 H, H4), 4.51 (d, *J*_2,3_ = 6.1 Hz, 1 H, H2), 4.84 (dd, *J*_3,2_ = 6.1, *J*_3,4_ = 1.0 Hz, 1 H, H3), 5.06 (bs, 1 H, H1_β_). ^13^C NMR (125 MHz): *δ* 24.8, 26.4, 65.2, 81.9, 86.5, 87.7, 106.7, 111.9. Anal. Calcd for C_8_H_14_O_5_: C, 50.52; H, 7.42. Found: C, 50.60; H, 7.40. The NMR spectral data were identical to values in the literature [19].
*Preparation of* 5*-O-tert-butyldiphenylsilyl-*2,3*-O-isopropylidene-d-ribofuranose* (**3**): TEA (0.28 mL, 2.0 mmol), DMAP (0.024 g, 0.2 mmol), and TBDPSCl (0.26 mL, 1.5 mmol) at 0 °C were added to a magnetically stirred solution of **2** (0.190 g, 1.0 mmol) in anhydrous CH_2_Cl_2_ (5.0 mL). The mixture was stirred at room temperature for 4 h. As soon as the starting compound **2** was completely consumed (TLC monitoring), the organic layer was washed with brine and dried (Na_2_SO_4_). The evaporation of the solvent under reduced pressure gave a crude residue that was purified by silica gel column chromatography (hexane-ethyl acetate 97:3 (*v*/*v*)) to give the oily product **3** as a yellowish syrup (0.386 g, 0.90 mmol, 90%). The product was obtained as a mixture of both anomers, but the βanomer was the main product (α:β, 1:1.9). ^1^H NMR (500 MHz): *δ* 1.08 (s, 9H_α_, CH_3_), 1.12 (s, 9H_β_,CH_3_), 1.34 (s, 3H_β_,CH_3_), 1.42 (s, 3H_α_ CH_3_), 1.50 (s, 3H, 3H_β_,CH_3_), 1.58 (s, 3H_α_, CH_3_), 3.71–3.63 (m, 2H, H5a_α+β_), 3.87–3.81 (m, 2H, H5b_α+β_), 3.96 (d, *J*_OH,1α_ = 11.4 Hz, 1H, OH_α_), 4.18 (m, 1H, H4_α_), 4.31 (m, 1H, H4 _β_),4.51 (d, *J*_OH,1β_ = 10.6 Hz, 1H, OH_β_), 4.63 (d, *J*_2,3_ = 5.9 Hz, 1H, H2_β_), 4.68 (dd, *J*_2,3_ = 6.2 Hz, *J*_2,1_ = 4.0 Hz, 1H, H2_α_), 4.74 (d, *J*_3,2_ = 5.9 Hz, 1H, H3_β_), 4.80 (d, *J*_3,2_ = 6.2 Hz, 1H, H3_α_), 5.37 (d, *J*_1,OH_ = 10.6 Hz, 1H, H1_β_), 5.64 (dd, *J*_1,OH_ = 11.4 Hz, *J*_1,2_ = 4.0 Hz, 1H, H1_α_), 7.53–7.64 (m, 20H, Ar_α+β_); ^13^C NMR (125 MHz): *δ* 19.1, 22.6, 25.0, 26.5, 27.0, 65.5, 66.1, 81.6, 87.1, 87.4, 98.0, 103.4, 127.7, 127.9, 128.0, 129.7, 130.2, 130.4, 134.8, 135.6, 135.7. Anal. Calcd for C_24_H_32_O_5_Si: C, 67.26; H, 5.53. Found: C, 67.31; H, 5.63.
*Preparation of* 5*-O-tert-butyldiphenylsilyl-*2,3*-O-isopropylidene-d-ribitol* (**4**): To a magnetically stirred solution of **3** (0.429 g, 1.0 mmol) in MeOH (10.0 mL), NaBH_4_ (0.113 g, 4.0 mmol) at 0 °C was added. The mixture was stirred at room temperature for 2.5 h. As soon as the starting compound **3** was completely consumed (TLC monitoring), the solvent was evaporated under reduced pressure and replaced with AcOEt. The organic layer was washed with brine and dried (Na_2_SO_4_). The evaporation of the solvent under reduced pressure gave the pure product **4** as a colorless syrup (0.430 g, 1.0 mmol, >99%). ^1^H NMR (400 MHz): *δ* 1.10 (s, 9H, CH_3_), 1.31 (s, 3H, CH_3_), 1.33 (s, 3H, CH_3_), 3.10 (m, 2H, H4 and OH), 3.77–3.93 (m, 4H, H1 and H5), 4.14 (m, 1H, H2), 4.38 (m, 1H, H3), 7.40–7.46 (m, 6H, Ar), 7.67–7.70 (m, 4H, Ar); ^13^C NMR (125 MHz): *δ* 19.3, 25.2, 26.9, 27.7, 60.9, 65.3, 69.7, 76.5, 77.6, 108.5, 127.8, 130.0, 132.8, 132.9, 135.6. Anal. Calcd for C_24_H_34_O_5_Si: C, 66.94; H, 7.96. Found: C, 66.91; H, 7.93.
*Preparation of* 5-*O-tert-butyldiphenylsilyl-*2,3*-O-isopropylidene-*1,4-*di-O-methanesulfonyl-d-ribitol* (**5**): DMAP (0.002 g, 0.02 mmol), TEA (1.1 mL, 8.0 mmol), and mesylchloride (0.309 mL, 4.0 mmol) at 0 °C were added to a magnetically stirred solution of **4** (0.430 g, 1.0 mmol) in CH_2_Cl_2_ (14.0 mL), and the mixture was stirred at room temperature overnight. The organic layer was washed with brine and dried (Na_2_SO_4_). The evaporation of the solvent under reduced pressure gave a crude residue that was purified by silica gel column chromatography (hexane-ethyl acetate 6:4 (*v*/*v*)) to give the oily product **5** as a yellowish syrup (0.575 g, 0.98 mmol, 98%). ^1^H NMR (500 MHz): *δ* 1.11 (s, 9H, CH3), 1.38 (s, 3H, CH_3_), 1.43 (s, 3H, CH_3_), 3.06 (s, 6H, CH_3_), 4.00 (dd, *J*_5a,5b_ = 12.2 Hz, *J*_5a,4_ = 3.8 Hz, 1H, H5a), 4.07 (dd, *J*_5b,5a_ = 12.2 Hz, *J*_5b,4_ = 2.7 Hz, 1H, H5b), 4.34 (dd, *J*_1a,1b_ = 10.4 Hz, *J*_1a,2_ = 6.4 Hz, 1H, H1a), 4.46–4.54 (m, 3H, H1b, H2 and H3), 4.85 (m, 1H, H4), 7.40–7.49 (m, 6H, Ar), 7.73–7.67 (m, 4H, Ar); ^13^C NMR (125 MHz): *δ* 19.3, 25.4, 26.8, 27.4, 37.5, 39.3, 63.1, 68.3, 73.8, 74.9, 79.2, 109.5, 127.9, 130.1, 132.4, 132.5, 135.6. Anal. Calcd for C_26_H_38_O_9_S_2_Si: C, 53.22; H, 6.53. Found: C, 53.23; H, 6.43.
*Preparation of* 5*-O-tert-butyldiphenylsilyl-*1,4-*deoxy-*2,3*-O-isopropylidene-*4*-seleno-l-lyxose* (**6**): NaBH_4_ was added to a magnetically stirred suspension of selenium powder (0.166 g, 2.1 mmol) in dry ethanol (13 mL) under N_2_ atmosphere, and the mixture was stirred at room temperature until the color of the mixture changed from black to colorless. Then, compound **5** (0.586 g, 1 mmol) in dry THF (8 mL) was added to the mixture, which was heated at 60 °C overnight. Then, the solvent was removed and replaced with AcOEt. Next, the organic layer was washed with brine and dried (Na_2_SO_4_). The evaporation of the solvent under reduced pressure gave the pure seleno derivative **6** as a pale-yellow oil (0.470 g, 0.99 mmol, 99%). ^1^H NMR (500 MHz): *δ* 1.08 (s, 9H, CH_3_), 1.30 (s, 3H, CH_3_), 1.48 (s, 3H, CH_3_), 2.97 (dd, *J*_1a,1b_ = 12.2 Hz, *J*_1a,2_ = 1.5 Hz, 1H, H1a), 3.03 (dd, *J*_1b,1a_ = 12.2 Hz, *J*_1b,2_ = 4.7 Hz, 1H, H1b), 3.77 (m, 1H, H4), 3.93 (dd, *J*_5a,5b_ = 10.2 Hz, *J*_5a,4_ = 7.9 Hz, 1H, H5a), 4.15 (dd, *J*_5b,5a_ = 10.2 Hz, *J*_5b,4_ = 7.1 Hz, 1H, H5b), 4.77 (m, 1H, H2), 4.94 (dd, *J*_3,2_ = 5.3 Hz, *J*_3,4_ = 4.5 Hz, 1H, H3), 7.37–7.47 (m, 6H, Ar), 7.72 (ddd, *J* = 11.0, 7.9, 1.4 Hz, 4H, Ar); ^13^C NMR (125 MHz): *δ* 19.3, 24.7, 26.1, 26.8, 29.8, 50.5, 63.0, 83.3, 84.9, 110.3, 127.6, 127.7, 129.6, 129.7, 133.5, 133.6, 135.7, 135.7. Anal. Calcd for C_24_H_32_O_3_SeSi: C, 60.62; H, 6.78. Found: C, 60.59; H, 6.83.
*Under the above conditions, 5-O-tert-butyldiphenylsilyl-1,4-deoxy-2,3-O-isopropylidene-4-seleno-d-ribose* (**10**) *was obtained as a pale-yellow oil (96%) starting from the C4 isomer of compound* **5**: ^1^H NMR (400 MHz): *δ* 1.09 (s, 9H, CH_3_), 1.33 (s, 3H, CH_3_), 1.54 (s, 3H, CH_3_), 2.96 (dd, *J*_1a,1b_ = 11.8 Hz, *J*_1a,2_ = 2.3 Hz, 1, H, H1a), 3.20 (dd, *J*_1b,1a_ = 11.8 Hz, *J*_1b,2_ = 5.1 Hz, 1, H, H1b), 3.67 (ddt, *J*_4,5a_ = 7.3 Hz, *J*_4,5b_ = 5.3 Hz, *J*_4,3_ = 1.8 Hz, 1H, H4), 3.74 (dd, *J*_5a,5b_ = 10.5 Hz, *J*_5a,4_ = 7.0 Hz, 1H, H5a), 3.88 (dd, *J*_5b,5a_ = 10.5 Hz, *J*_5b,4_ = 5.4 Hz, 1H, H5b), 4.80 (dd, *J*_3,2_ = 5.3 Hz, *J*_3,4_ = 1.8 Hz, 1H, H3), 4.86 (ddd, *J*_2,1b_ = 5.5 Hz, *J*_2,3_ = 5.4 Hz, *J*_2,1a_ = 2.3 Hz, 1H, H2), 7.38–7.47 (m, 6H, Ar), 7.65–7.72 (m, 4H, Ar); ^13^C NMR (100 MHz): *δ* 19.2, 24.7, 26.9, 29.6, 50.1, 64.5, 85.1, 87.3, 110.3, 127.7, 129.8, 133.1, 135.6. Anal. Calcd for C_24_H_32_O_3_SeSi: C, 60.62; H, 6.78. Found: C, 60.63; H, 6.79.
*Preparation of* 1,4*-deoxy-*2,3*-O-isopropylidene-*4*-seleno-l-lyxose* (**7**): A solution of tetrabutylammonium fluoride (1.7 mL of a 1.1 M solution in THF, 2.0 mmol) was added to a magnetically stirred solution of seleno derivative **6** (0.475 g, 1.0 mmol) in anhydrous THF (7.0 mL) at 0 °C under N_2_, and the mixture was stirred at room temperature for 1 h. The evaporation of the solvent under reduced pressure gave a crude residue that was purified by silica gel column chromatography (hexane-ethyl acetate 6:4 (*v*/*v*)) to give the pure product **7** as a crystalline white solid (0.216 g, 0.91 mmol, 91%). ^1^H NMR (500 MHz): *δ* 1.33 (s, 3H, CH_3_), 1.53 (s, 3H, CH_3_), 2.53 (brs, 1H, OH), 2.99–3.05 (m, 2H, H1), 3.66 (dt, *J*_4,5b_ = 7.5 Hz, *J*_4,5a_ = 5.0 Hz, *J*_4,3_ = 4.2 Hz, 1H, H4), 3.91 (dd, *J*_5a,5b_ = 11.5 Hz, *J*_5a,4_ = 5.0 Hz, 1H, H5a), 4.00 (dd, *J*_5b,5a_ = 11.5 Hz, *J*_5b,4_ = 7.5 Hz, 1H, H5b), 4.87 (m, 1H, H3), 4.94 (ddd, *J*_2,3_ = 6.1 Hz, *J*_2,1b_ = 4.1 Hz, *J*_2,1a_ = 2.0 Hz, 1H, H2); ^13^C NMR (125 MHz): *δ* 24.5, 26.1, 29.0, 48.2, 62.2, 85.0, 85.1, 111.5. Anal. Calcd for C_8_H_14_O_3_Se: C, 40.52; H, 5.95. Found: C, 40.49; H, 5.96.
*Under the above conditions, 1,4-deoxy-2,3-O-isopropylidene-4-seleno-d-ribose* (**11**) *was obtained as an amorphous solid (90%) starting from the C4 isomer of compound* **10**. ^1^H NMR (500 MHz): *δ* 1.33 (s, 3H, CH_3_), 1.54 (s, 3H, CH_3_), 3.02 (dd, *J*_1a,1b_ = 12.0, *J*_1a,2_ = 2.1 Hz, 1H, H1a), 3.22 (dd, *J*_1b,1a_ = 12.0 Hz, *J*_1b,2_ = 5.2, 1H, H1b), 3.62–3.75 (m, 3H, H4 and H5), 4.73 (dd, *J*_3,2_ = 5.5 Hz, *J*_3,4_ = 1.4 Hz, 1H, H3), 4.99 (ddd, *J*_2,3_ = 5.5 Hz, *J*_2,1b_ = 5.2 Hz, *J*_2,1a_ = 2.1 Hz, 1H, H2); ^13^C NMR (125 MHz): *δ* 24.7, 26.8, 29.4, 52.2, 64.1, 85.1, 87.7, 111.6. Anal. Calcd for C_8_H_14_O_3_Se: C, 40.52; H, 5.95. Found: C, 40.55; H, 5.93.
*Preparation of monoacetyl hydroquinone*: DMAP (0.01 g, 0.091 mmol) was added to a magnetically stirred solution of hydroquinone (0.100 g, 0.908 mmol) in acetic anhydride (1.09 mmol, 0.103 mL) and dry pyridine (0.90 mL) under N_2_. The reaction mixture was stirred overnight at room temperature and then diluted in ethyl acetate (50 mL). The resulting solution was washed with HCl (1 M, 50 mL × 4), NH_4_Cl (50 mL), and brine and dried (Na_2_SO_4_). The evaporation of the solvent under reduced pressure gave a crude residue that was purified by silica gel column chromatography (CHCl_3_) to give the pure monoacetylated hydroquinone as a crystalline white solid (0.125 g, 0.82 mmol, 70%). The NMR spectral data were identical to the values in the literature [25].
*Mitsunobu Reaction: Preparation of glycoconjugates. Typical Procedure*: DIAD (0.303 mL, 1.5 mmol) was added to a magnetically stirred solution of TPP (0.393 g, 1.5 mmol) in anhydrous THF (2.3 mL) at 0 °C under N_2_. After 15 min, a solution containing seleno derivative **7** (0.200 g, 1.0 mmol) and monoacetylated hydroquinone (0.270 g, 1.5 mmol) in anhydrous THF (3.0 mL) was added dropwise. The reaction mixture was stirred at room temperature for three days. The solvent was evaporated under reduced pressure and replaced with AcOEt. The organic layer was washed with brine and dried (Na_2_SO_4_). The evaporation of the solvent under reduced pressure gave a crude residue that was purified by silica gel column chromatography (hexane-diethyl ether 7:3 (*v*/*v*)) to give the pure product **8** as a pale yellow-syrup (0.160 g, 0.43 mmol, 43%). ^1^H NMR (500 MHz): *δ* 1.36 (s, 3H, CH_3_), 1.57 (s, 3H, CH_3_), 2.30 (s, 3H, CH_3_), 3.08 (dd, *J*_1a,1b_ = 12.0 Hz, *J*_1a,2_ = 1.5 Hz, 1H, H1a), 3.34 (dd, *J*_1b,1a_ = 12.0 Hz, *J*_1b,2_ = 5.0 Hz, 1H, H1b), 3.85 (m, 1H, H4), 4.03 (dd, *J*_5a,5b_ = 9.5 Hz, *J*_5a,4_ = 8.0 Hz, 1H, H5a), 4.21 (dd, *J*_5b,5a_ = 9.5 Hz, *J*_5b,4_ = 5.5 Hz, 1H, H5b), 4.90 (dd, *J*_3,2_ = 5.5 Hz, *J*_3,4_ = 1.5 Hz, 1H, H3), 5.05 (dt, *J*_2,3_ = 5.5 Hz, *J*_2,1b_ = 5.0 Hz, *J*_2,1a_ = 2.0 Hz, 1H, H2), 6.90 (d, *J* = 9.0 Hz, 2H, Ar), 7.00 (d, *J* = 9.0 Hz, 2H, Ar); ^13^C NMR (125 MHz): *δ* 21.0, 24.7, 26.7, 30.2, 47.0, 70.9, 85.2, 87.6, 110.6, 115.3, 122.4, 144.6, 156.0, 169.8. Anal. Calcd for C_16_H_20_O_5_Se: C, 51.76; H, 5.43. Found: C, 51.66; H, 5.48.
*Under the above conditions, 5-(4-hydroxyphenoxy)-1,4-deoxy-2,3-O-isopropylidene-4-seleno-d-ribose* (**12**) *was obtained as oil (13%) starting from the compound* **7** *and hydroquinone:* ^1^H NMR (500 MHz): *δ* 1.36 (s, 3H, CH_3_), 1.56 (s, 3H, CH_3_), 3.06 (dd, *J*_1a,1b_ = 11.9 Hz, *J*_1a,2_ = 1.9 Hz, 1H, H1a), 3.33 (dd, *J*_1b,1a_ = 11.9 Hz, *J*_1b,2_ = 5.1 Hz, 1H, H1b), 3.83 (m, 1H, H4), 3.99 (dd, *J*_5a,5b_ = 9.9 Hz, *J*_5a,4_ = 7.8 Hz, 1H, H5a), 4.16 (dd, *J*_5b,5a_ = 9.9 Hz, *J*_5b,4_ = 5.2 Hz, 1H, H5b), 4.91 (dd, *J*_3,2_ = 5.6 Hz, *J*_3,4_ = 1.7 Hz, 1H, H3), 5.05 (dt, *J*_2,3_ = 5.6 Hz, *J*_2,1b_ = 5.3 Hz, *J*_2,1a_ = 1.9 Hz, 1H, H2), 6.77 (d, *J* = 9.2 Hz, 2H, Ar), 6.80 (d, *J* = 9.3 Hz, 2H, Ar); ^13^C NMR (125 MHz): *δ* 24.7, 26.7, 30.2, 47.2, 71.3, 85.2, 87.7, 110.6, 115.9, 116.1, 150.0, 152.5. Anal. Calcd for C_14_H_18_O_4_Se: C, 51.07; H, 5.51. Found: C, 51.10; H, 5.50.
*Under the above conditions, (E)-5-((3-(3,4-dihydroxyphenyl)acryloyl)oxy)-1,4-deoxy-2,3-O-isopropylidene-4-seleno-d-ribose* (**13**) *was obtained as oil (70%) starting from the compound* **7** *and caffeic acid:* ^1^H NMR (500 MHz): *δ* 1.34 (s, 3H, CH_3_), 1.54 (s, 3H, CH_3_), 3.06 (dd, *J*_1a,1b_ = 12.1 Hz, *J*_1a,2_ = 1.7 Hz, 1H, H1a), 3.26 (dd, *J*_1b,1a_ = 12.1 Hz, *J*_1b,2_ = 4.9 Hz, 1H, H1b), 3.76 (m, 1H, H4), 4.26 (dd, *J*_5a,5b_ = 11.6 Hz, *J*_5a,4_ = 8.9 Hz, 1H, H5a), 4.37 (dd, *J*_5b,5a_ = 11.6 Hz, *J*_5b,4_ = 5.8 Hz, 1H, H5b), 4.77 (dd, *J*_3,2_ = 5.5 Hz, *J*_3,4_ = 1.6 Hz, 1H, H3), 5.03 (ddd, *J*_2,3_ = 5.7 Hz, *J*_2,1b_ = 5.0 Hz, *J*_2,1a_ = 2.0 Hz, 1H, H2), 6.00 (brs, 2H, OH), 6.24 (d, *J* = 15.9 Hz, 1H, H2′), 6.87 (d, *J* = 8.2 Hz, 1H, H8′), 7.00 (dd, *J* = 8.2 Hz, *J* = 1.5 Hz, 1H, H9′), 7.07 (d, *J* = 1.5 Hz, 1H, H5′), 7.58 (d, *J* = 15.9 Hz, 1H, H3′); ^13^C NMR (125 MHz): *δ* 24.7, 26.7, 29.7, 46.6, 65.7, 85.0, 87.3, 110.9, 114.5, 115.1, 115.6, 122.7, 127.6, 143.8, 145.5, 146.3, 167.0. Anal. Calcd for C_17_H_20_O_6_Se: C, 51.14; H, 5.05. Found: C, 51.19; H, 5.03.
*Under the above conditions, (E)-5-((3-(4-hydroxy-3-methoxyphenyl)acryloyl)oxy)-1,4-deoxy-2,3-O-isopropylidene-4-seleno-d-ribose* (**14**) *was obtained as oil (68%) starting from the compound* **7** *and ferulic acid:* ^1^H NMR (500 MHz, acetone-d_6_): *δ* 1.30 (s, 3H, CH_3_), 1.46 (s, 3H, CH_3_), 2.99 (dd, *J*_1a,1b_ = 11.9 Hz, *J*_1a,2_ = 1.3 Hz, 1H, H1a), 3.37 (dd, *J*_1b,1a_ = 11.9 Hz, *J*_1b,2_ = 4.9 Hz, 1H, H1b), 3.76 (ddd, *J*_4,5a_ = 8.7 Hz, *J*_4,5b_ = 7.4 Hz, *J*_4,3_ = 1.3 Hz, 1H, H4), 3.95 (s, 3H, CH_3_), 4.26 (dd, *J*_5a,5b_ = 11.4 Hz, *J*_5a,4_ = 8.8 Hz, 1H, H5a), 4.36 (dd, *J*_5b,5a_ = 11.4 Hz, *J*_5b,4_ = 6.8 Hz, 1H, H5b), 4.86 (dd, *J*_3,2_ = 5.8 Hz, *J*_3,4_ = 1.5 Hz, 1H, H3), 5.11 (ddd, *J*_2,3_ = 6.1, *J*_2,1b_ = 5.9 Hz, *J*_2,1a_ = 1.9 Hz, 1H, H2), 6.46 (d, *J* = 16.0 Hz, 1H, H2′), 6.90 (dd, *J* = 8.0 Hz, 1H, H8′), 7.18 (dd, *J* = 8.5 Hz, *J* = 1.9 Hz, 1H, H9′), 7.39 (d, *J* = 1.5 Hz, 1H, H5′), 7.66 (d, *J* = 16.0 Hz, 1H, H3′); ^13^C NMR (125 MHz): *δ* 24.7, 26.7, 29.3, 46.7, 56.0, 65.6, 85.0, 87.3, 109.4, 110.8, 114.8, 114.8, 123.3, 126.8, 145.7, 146.8, 148.2, 166.9. Anal. Calcd for C_18_H_22_O_6_Se: C, 52.31; H, 5.37. Found: C, 52.25; H, 5.43.
*Under the above conditions, 5-(4-((1E,6E)-7-(4-hydroxy-3-methoxyphenyl)-3,5-dioxohepta-1,6-dien-1-yl)-2-methoxyphenoxy)-1,4-deoxy-2,3-O-isopropylidene-4-seleno-d-ribose* (**15**) *was obtained as oil (30%) starting from the compound* **7** *and curcumin:* ^1^H NMR (400 MHz): *δ* 1.36 (s, 3H, CH_3_), 1.57 (s, 3H, CH_3_), 3.04 (dd, *J*_1a,1b_ = 12.0 Hz, *J*_1a,2_ = 1.9 Hz, 1H, H1a), 3.55 (dd, *J*_1b,1a_ = 12.0 Hz, *J*_1b,2_ = 5.0 Hz, 1H, H1b), 3.93–3.86 (m, 4H, H4 and CH_3_), 3.96 (s, 3H, CH_3_), 4.16 (dd, *J*_5a,5b_ = 9.7 Hz, *J*_5a,4_ = 8.0 Hz, 1H, H5a), 4.33 (dd, *J*_5b,5a_ = 9.7 Hz, *J*_5b,4_ = 5.2 Hz, 1H, H5b), 4.95 (dd, *J*_3,2_ = 5.6 Hz, *J*_3,4_ = 1.8 Hz, 1H, H3), 5.11 (ddd, *J*_2,3_ = 5.6 Hz, *J*_2,1b_ = 5.0 Hz, *J*_2,1a_ = 1.9 Hz, 1H, H2), 5.82 (m, 2H, H10′), 5.98 (bs, 1H, OH), 6.52–6.46 (m, 2H, H8′ and H12′), 6.84 (d, *J* = 8.3 Hz, 1H, H6′), 6.94 (d, *J* = 8.3 Hz, 1H, H18′), 7.05 (d, *J* = 1.7 Hz, 1H, H15′), 7.07 (d, *J* = 1.9 Hz, 1H, H3′), 7.14–7.09 (m, 2H, H5′ and H19′), 7.65–7.561 (m, 2H, H7′ and H13′); ^13^C NMR (125 MHz): *δ* 24.6, 26.7, 31.1, 47.1, 56.0, 72.1, 85.6, 88.2, 101.2, 109.6, 110.4, 113.1, 114.8, 121.8, 122.3, 122.9, 129.0, 140.2, 140.6, 146.8, 147.9, 149.7, 182.9, 183.6. Anal. Calcd for C_29_H_32_O_8_Se: C, 59.29; H, 5.49. Found: C, 59.19; H, 5.55.
*Removal of the O-isopropylidene group. Typical Procedure*: A solution (3.3 mL) of CH_3_COOH/H_2_O (8:2, *v*/*v*) was added to compound **12** (0.329 g, 1.0 mmol). The mixture was stirred at 80 °C for 2 h. Then, the solvent was evaporated under reduced pressure; next, the organic layer was washed with diethylether. The crude residue was purified to afford compound **12a** as an amorphus solid (0.205 g, 0.8 mmol, 71%). ^1^H NMR (400 MHz, acetone-*d_6_*): *δ* 2.82 (dd, *J*_1a,1b_ = 10.0 Hz, *J*_1a,2_ = 5.1 Hz, 1H, H1a), 3.02 (dd, *J*_1b,1a_ = 10.0 Hz, *J*_1b,2_ = 5.0 Hz, 1H, H1b), 3.77 (m, 1H, H4), 3.99 (dd, *J*_5a,5b_ = 9.7 Hz, *J*_5a,4_ = 8.3 Hz, 1H, H5a), 4.10 (m, 1H, H3), 4.35 (dd, *J*_5b,5a_ = 9.7 Hz, *J*_5b,4_ = 5.9 Hz, 1H, H5b), 4.40 (m, 1H, H2), 6.75 (d, *J* = 9.2 Hz, 2H, Ar), 6.79 (d, *J* = 9.2 Hz, 2H, Ar); ^13^C NMR (100 MHz, acetone-*d_6_*): *δ* 23.9, 42.6, 72.4, 76.0, 78.4, 115.8, 151.6, 152.0. Anal. Calcd for C_11_H_14_O_4_Se: C, 45.69; H, 4.88. Found: C, 45.73; H, 4.83.
*Under the above conditions, (E)-5-((3-(3,4-dihydroxyphenyl)acryloyl)oxy)-1,4-deoxy-4-seleno-d-ribose* (**13a**) *was obtained as an amorphous solid (68%) starting from the compound* **13**: ^1^H NMR (500 MHz, acetone-d_6_): *δ* 2.84 (dd, *J*_1a,1b_ = 9.9 Hz, *J*_1a,2_ = 5.3 Hz, 1H, H1a), 3.06 (dd, *J*_1b,1a_ = 9.9 Hz, *J*_1b,2_ = 5.1 Hz, 1H, H1b), 3.74 (m, 1H, H4), 4.10 (dd, *J*_3,4_ = 5.9 Hz, *J*_3,2_ = 3.2 Hz, 1H, H3), 4.22 (dd, *J*_5a,5b_ = 11.3 Hz, *J*_5a,4_ = 7.6 Hz, 1H, H5a), 4.43 (ddt, *J*_2,1a_ = 5.3 Hz, *J*_2,1b_ = 5.1 Hz, *J*_2,3_ = 3.1 Hz, 1H, H2), 4.57 (dd, *J*_5b,5a_ = 11.3 Hz, *J*_5b,4_ = 6.6 Hz, 1H, H5b), 6.32 (d, *J* = 15.9 Hz, 1H, H2′), 6.90 (d, *J* = 8.2 Hz, 1H, H5′), 7.09 (dd, *J* = 8.2 Hz, *J* = 2.0 Hz, 1H, H9′), 7.19 (d, *J* = 2.0 Hz, 1H, H8′), 7.58 (d, *J* = 15.6 Hz, 1H, H3′); ^13^C NMR (125 MHz, acetone-d_6_): *δ* 24.1, 41.9, 66.5, 76.0, 78.4, 114.4, 115.5, 121.7, 126.7, 145.2, 145.4, 148.0, 166.2. Anal. Calcd for C_14_H_16_O_6_Se: C, 46.81; H, 4.49. Found: C, 46.81; H, 4.45.
*Under the above conditions, (E)-5-((3-(4-hydroxy-3-methoxyphenyl)acryloyl)oxy)-1,4-deoxy-4-seleno-d-ribose* (**14a**), *an amorphous solid, was obtained (80%) starting from the compound* **14**: ^1^H NMR (500 MHz, acetone-d_6_): *δ* 2.85 (dd, *J*_1a,1b_ = 10.0 Hz, *J*_1a,2_ = 5.3 Hz, 1H, H1a), 3.06 (dd, *J*_1b,1a_ = 10.0 Hz, *J*_1b,2_ = 5.0 Hz, 1H, H1b), 3.74 (m, 1H, H4), 3.95 (s, 3H, CH_3_), 4.09 (m, 1H, H3), 4.22 (dd, *J*_5a,5b_ = 11.3 Hz, *J*_5a,4_ = 7.8 Hz, 1H, H5a), 4.43 (m, 1H, H2), 4.58 (dd, *J*_5b,5a_ = 11.3 Hz, *J*_5b,4_ = 6.5 Hz, 1H, H5b), 6.43 (d, *J* = 15.9 Hz, 1H, H2′), 6.90 (d, *J* = 8.1 Hz, 1H, H8′), 7.18 (dd, *J* = 8.2 Hz, *J* = 1.7 Hz, 1H, H9′), 7.39 (bs, 1H, H5′), 7.64 (d, *J* = 15.9 Hz, 1H, H3′); ^13^C NMR (125 MHz, acetone-d_6_): *δ* 24.1, 41.9, 55.5, 66.6, 76.0, 78.4, 110.4, 114.6, 115.2, 123.3, 126.5, 145.2, 145.3, 147.9, 149.3, 166.3. Anal. Calcd for C_15_H_18_O_6_Se: C, 48.27; H, 4.86. Found: C, 48.36; H, 4.82.

## 4. Conclusions

A new route for synthesizing unusual seleno-glyconjugates has been developed, exploiting a Mitsunobu reaction. Interestingly, the latter proceeded with high stereocontrol, showing a preferential bias for the introduction of the acceptor moiety, leading to a compound with the 3,4-*anti*-configuration not depending on the stereochemistry of the starting seleno-donor.

## Figures and Tables

**Table 1:** Vicinal coupling constants. Comparative analysis.

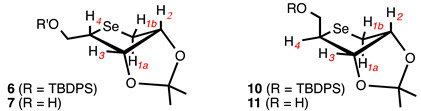				
**Compound**	**^3^**J*_2,1a_***	**^3^**J*_2,1b_***	**^3^**J*_3,2_***	**^3^**J*_3,4_***
**6**	1.5	4.7	5.3	4.5
**7**	2.0	4.1	6.1	4.2
**10**	2.3	5.1	5.3	1.8
**11**	2.1	5.2	5.5	1.4

**Table 2:** Scope with respect to polyphenolic substrates ^1^.

**Polyphenol**	**Glycoconjugate**	**Yield ^2^**
** 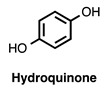 **	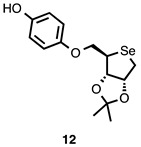	13 (14) ^3^
** 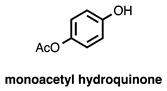 **	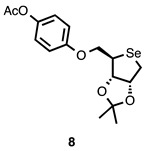	43 (44) ^3^
** 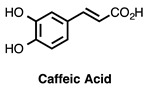 **	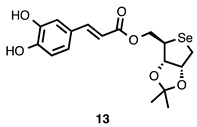	70 (72) ^3^
** 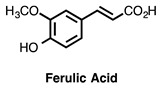 **	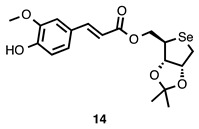	68 (67) ^3^
** 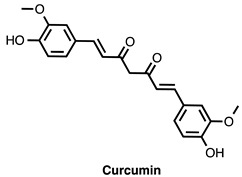 **	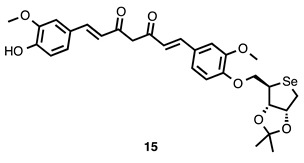	30 (28) ^3^

^1^ Reaction carried out on derivative **7** (1 eq), TPP (1.5 eq), diisopropyl azodicarboxyate (DIAD, 1.5 eq), and polyphenol (1.5 eq) in dry THF under N_2_ atmosphere for three days. ^2^ Percentage yield; isolated yield by column chromatography; double reaction products of the polyphenol were never detected. ^3^ Percentage yield; reaction starting from derivative **11** under same condition.

## Data Availability

The data presented in this study are available on request from the corresponding author.

## Supplementary Materials

The following are available online. ^1^H, ^13^C NMR spectra of products.

## Sample Availability

Not available.

## References

[1] Lin M.T., Beal M.F. (2006). Mitochondrial dysfunction and oxidative stress in neurodegenerative diseases. Nature.

[2] Barnham K.J., Masters C.L., Bush A.I. (2004). Neurodegenerative diseases and oxidative stress. Nat. Rev. Drug Discov..

[3] Cervellati C., Wood P.L., Romani A., Valacchi G., Squerzanti M., Sanz J.M., Ortolani B., Zuliani G. (2016). Oxidative challenge in Alzheimer’s disease: State of knowledge and future needs. J. Investig. Med..

[4] Gilgun-Sherki Y., Melamed E., Offen D.J. (2003). Antioxidant treatment in Alzheimer’s disease. J. Mol. Neurosci..

[5] Trippier P.C., Labby K.J., Hawker D.D., Mataka J.J., Silverman R.B. (2013). Target- and mechanism-based therapeutics for neurodegenerative diseases: Strength in numbers. J. Med. Chem..

[6] Nogueira C.W., Rocha J.B.T. (2011). Toxicology and pharmacology of selenium: Emphasis on synthetic organoselenium compounds. Arch. Toxicol..

[7] Moussaoui S., Obinu M.C., Daniel N., Reibaud M., Blanchard V., Imperato A. (2000). The antioxidant ebselen prevents neurotoxicity and clinical symptoms in a primate model of parkinson’s disease. Exp. Neurol..

[8] Aisen P.S., Gauthier S., Ferris S.H., Saumier D., Haine D., Garceau D., Duong A., Suhy J., Oh J., Lau W.C. (2011). Tramiprosate in mild-to-moderate Alzheimer’s disease—A randomized, double-blind, placebo-controlled, multi-centre study (the Alphase Study). Arch. Med. Sci..

[9] Green R.C., Schneider L.S., Amato D.A. (2009). Effect of Tarenflurbil on Cognitive Decline and Activities of Daily Living in Patients With Mild Alzheimer Disease. JAMA.

[10] Monteiro Espíndola K.M., Guimarães Ferreira R., Mosquera Narvaez L.E., Rocha Silva Rosario A.C., Machado da Silva A.H., Bispo Silva A.G., Oliveira Vieira A.P., Chagas Monteiro M. (2019). Chemical and pharmacological aspects of caffeic acid and its activity in hepatocarcinoma. Front. Oncol..

[11] Gomez-Bougie P., Halliez M., Maïga S., Godon C., Kervoëlen C., Pellat-Deceunynck C., Amiot M. (2015). Curcumin induces cell death of the main molecular myeloma subtypes, particularly the poor prognosis subgroups. Cancer Biol. Ther..

[12] Fan Z., Yao J., Li Y., Hu X., Shao H., Tian X. (2015). Anti-inflammatory and antioxidant effects of curcumin on acute lung injury in a rodent model of intestinal ischemia reperfusion by inhibiting the pathway of NF-Kb. Int. J. Clin. Exp. Pathol..

[13] Nobili A., Latagliata E.C., Viscomi M.T., Cavallucci V., Cutuli D., Giacovazzo G., Krashia P., Rizzo F.R., Marino R., Federici M. (2017). Dopamine neuronal loss contributes to memory and reward dysfunction in a model of Alzheimer’s disease. Nat. Commun..

[14] Jain V.K., Priyadarsini K.I. (2018). Organoselenium Compounds in Biology and Medicine: Synthesis, Biological and Therapeutic Treatments.

[15] Inagaki Y., Minakawa N., Matsuda A. (2007). Synthesis of 4′selenoribo nucleosides. Nucleic Acids Symp. Ser..

[16] Jayakanthan K., Johnston B.D., Pinto B.M. (2008). Stereoselective synthesis of 4′-selenonucleosides using the Pummerer glycosylation reaction. Carbohydr. Res..

[17] Jeong L.S., Tosh D.K., Kim H.O., Wang T., Hou X., Yun H.S., Kwon Y., Lee S.K., Choi J., Zhao L.X. (2008). First Synthesis of 4‘-selenonucleosides showing unusual southern conformation. Org. Lett..

[18] Yu J., Kim J.-H., Lee H.W., Alexander V., Ahn H.-C., Choi W.J., Choi J., Jeong L.S. (2013). New RNA purine building blocks, 4′-selenopurine nucleosides: First synthesis and unusual mixture of sugar puckerings. Chem. Eur. J..

[19] Pedatella S., Guaragna A., D’Alonzo D., De Nisco M., Palumbo G. (2006). Triphenylphosphine polymer-bound/iodine complex: A suitable reagent for the preparation of *o*-isopropylidene sugar derivatives. Synthesis.

[20] Caputo R., Kunz H., Mastroianni D., Palumbo G., Pedatella S., Solla F. (1999). Mild Synthesis of Protected α-D-Glycosyl Iodides. Eur. J. Org. Chem..

[21] Sapkota K., Roh E., Lee E., Ha E.-M., Yang J.-H., Lee E.-S., Kwon Y., Kim Y., Na Y. (2011). Synthesis and anti-melanogenic activity of hydroxyphenyl benzyl ether analogues. Bioorg. Med. Chem..

[22] Taniguchi T., Nakano K., Baba R., Monde K. (2017). Analysis of configuration and conformation of furanose ring in carbohydrate and nucleoside by vibrational circular dichroism. Org. Lett..

[23] Batra H., Moriarty R.M., Penmasta R., Sharma V., Stanciuc G., Staszewski J.P., Tuladhar S.M., Walsh D.A. (2006). A concise, efficient and production-scale synthesis of a protected l-lyxonolactone derivative:  An important aldonolactone core. Org. Process Res. Dev..

[24] Taniike H., Inagaki Y., Matsuda A., Minakawa N. (2011). Practical synthesis of 4’-selenopyrimidine nucleosides using hypervalent iodine. Tetrahedron.

[25] Cepanec I., Litvic M. (2008). Simple and efficient synthesis of arbutin. Arkivoc.

