# Supplementary material for: Stereoselective Synthesis of Selenium-Containing Glycoconjugates via the Mitsunobu Reaction
Source: Molecules. 2021 Apr 27;26(9):2541. doi: 10.3390/molecules26092541 (PMC8123786; doi:10.3390/molecules26092541)
Supplement: Supplementary file 1 [file molecules-26-02541-s001.zip › molecules-1170949-supplementary.pdf]

Stereoselective Synthesis of Polyphenol-Based Selenium-Containing Glycoconjugates *via* the Mitsunobu Reaction

Luigia Serpico[1], Mauro De Nisco[2,], Flavio Cermola[1], Michele Manfra[2], and Silvana Pedatella[1]*

Supporting Information

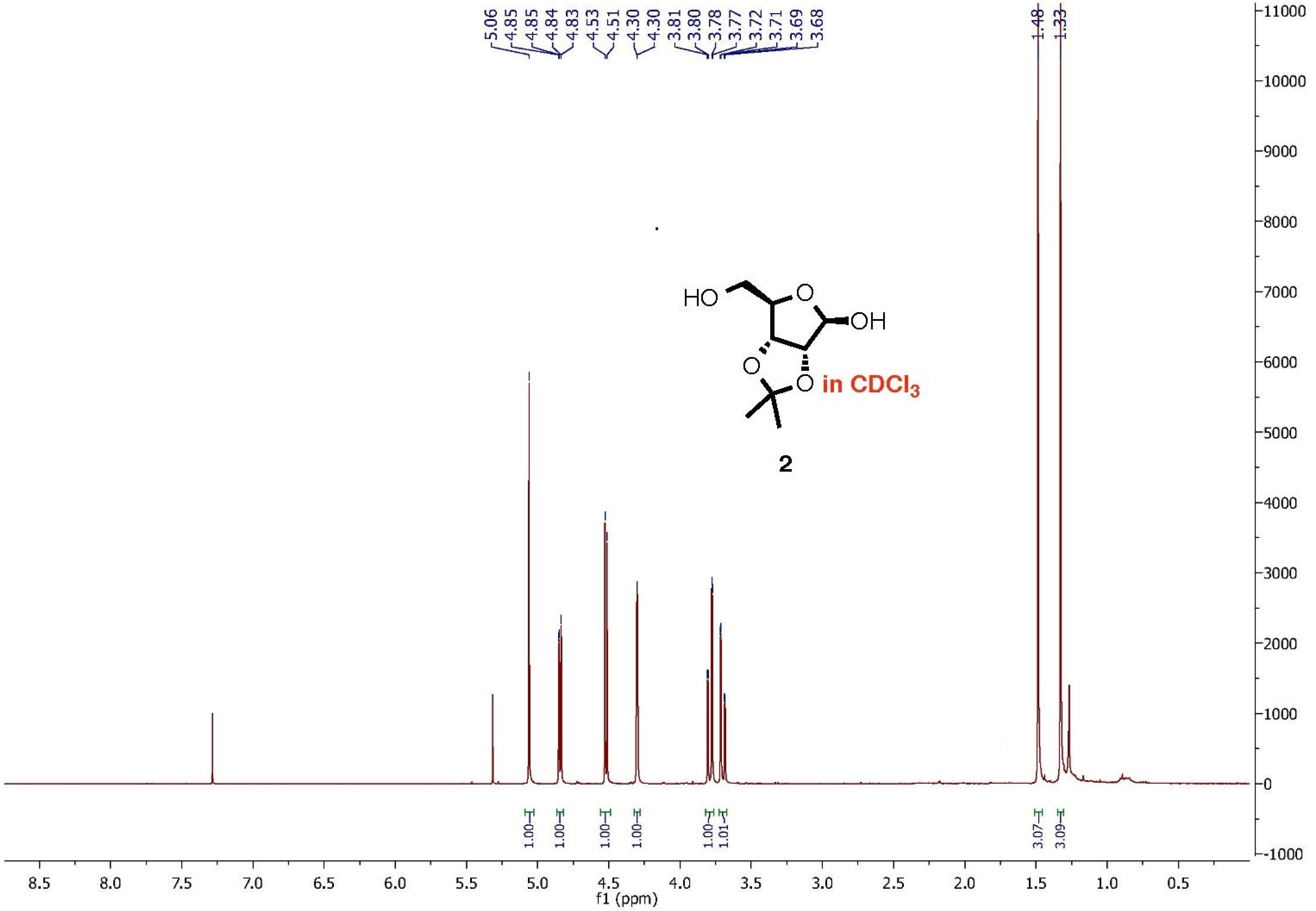

5.06 4.85 4.85 4.84 4.83 4.53 4.51 4.30 4.30 3.81 3.80 3.78 3.77 3.72 3.71 3.69 3.68
1.48 1.33
HO
O
OH
O
O
in $CDCl_3$
2
1.00 1.00 1.00 1.00 1.00 1.01 3.07 3.09
f1 (ppm)

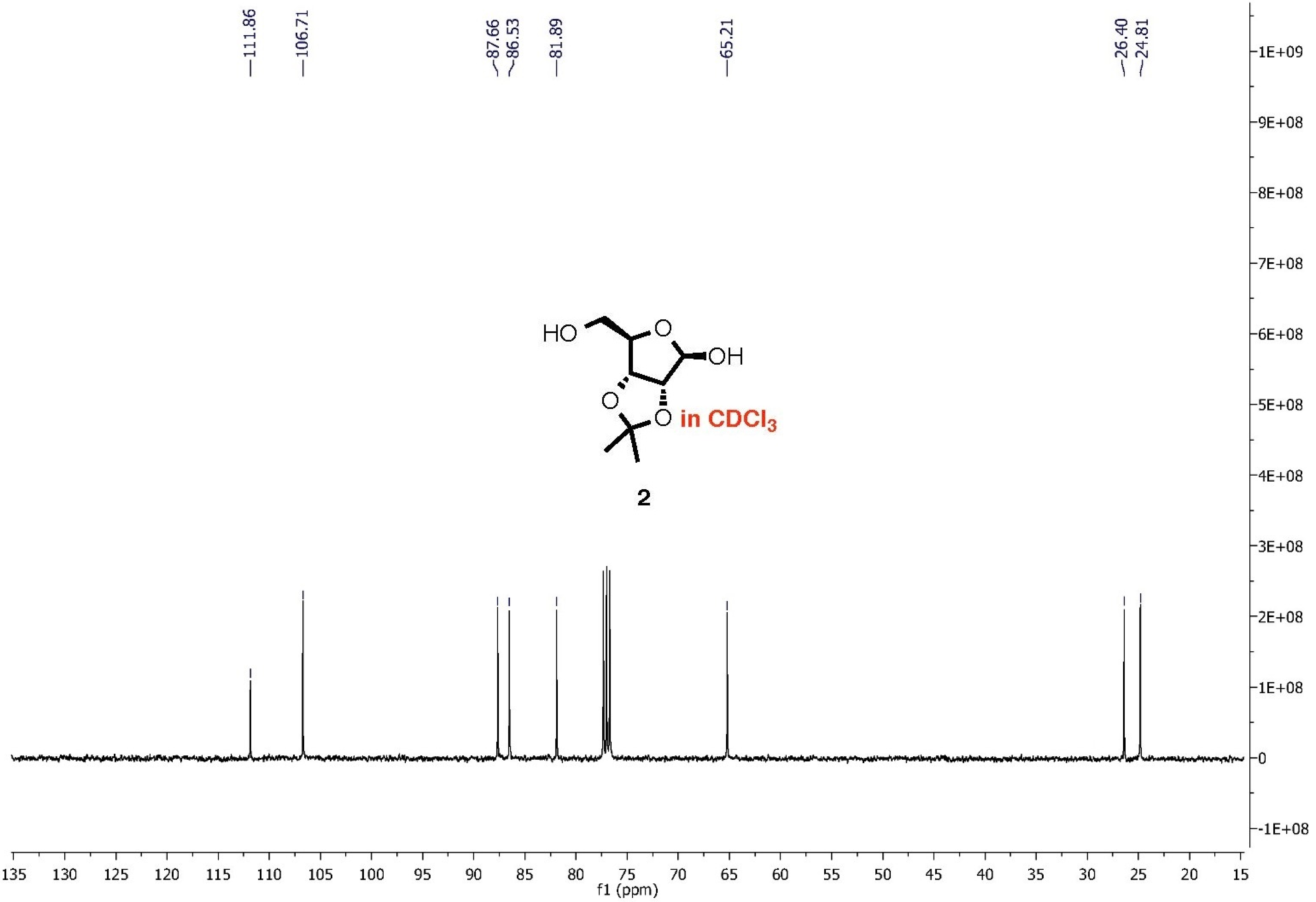
111.86
106.71
87.66
86.53
81.89
65.21
26.40
24.81
HO
O
OH
O
O
in $CDCl_3$
2
1E+09
9E+08
8E+08
7E+08
6E+08
5E+08
4E+08
3E+08
2E+08
1E+08
0
-1E+08
135 130 125 120 115 110 105 100 95 90 85 80 75 70 65 60 55 50 45 40 35 30 25 20 15
f1 (ppm)

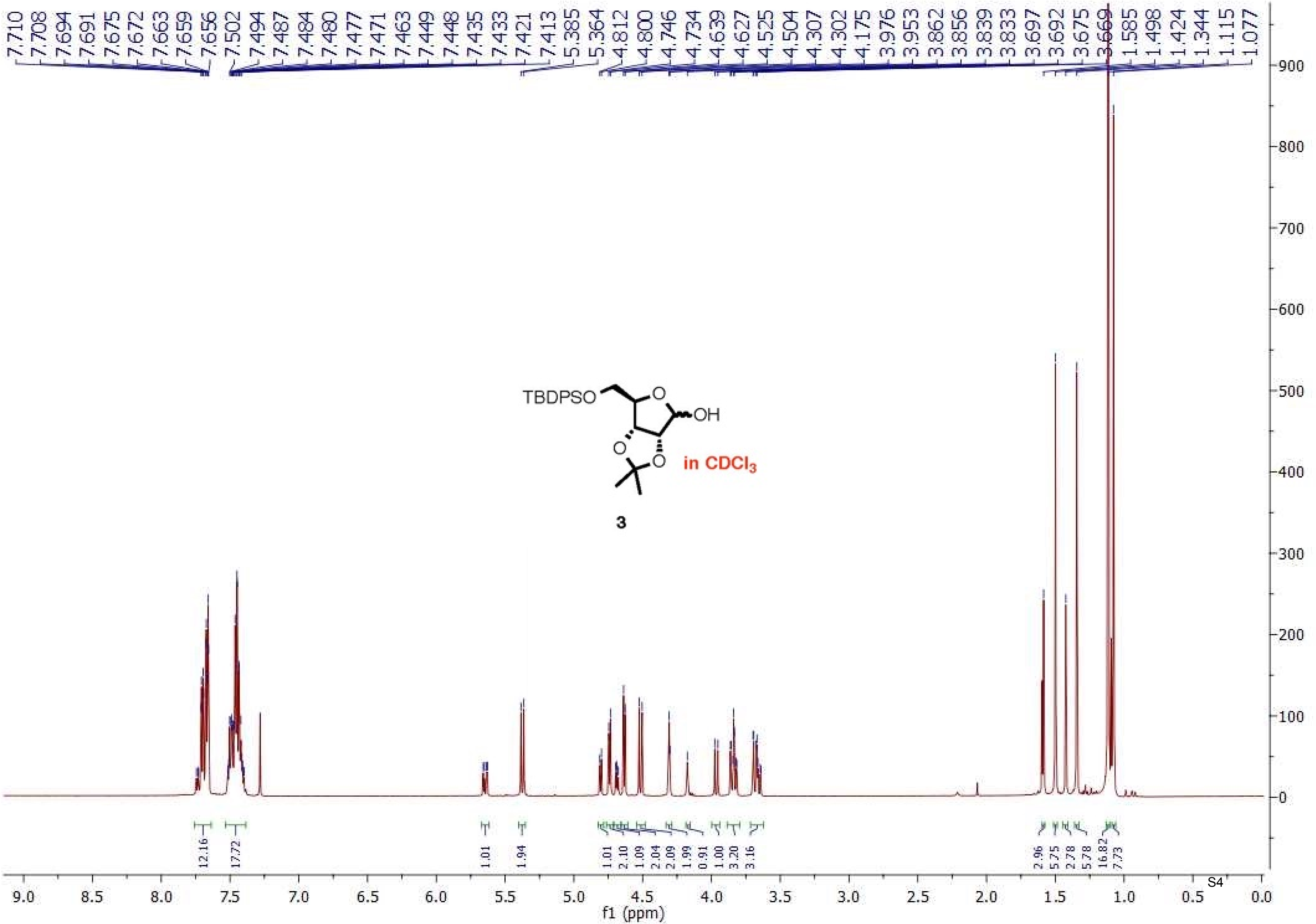
7.710 7.708 7.694 7.691 7.675 7.672 7.663 7.659 7.656 7.502 7.494 7.487 7.484 7.480 7.477 7.471 7.463 7.449 7.448 7.435 7.433 7.421 7.413 5.385 5.364 4.812 4.800 4.746 4.734 4.639 4.627 4.525 4.504 4.307 4.302 4.175 3.976 3.953 3.862 3.856 3.839 3.833 3.697 3.692 3.675 3.669 1.585 1.498 1.424 1.344 1.115 1.077
TBDPSO
OH
O
in $CDCl_3$
3
12.16 17.72 1.01 1.94 1.01 2.10 1.09 2.04 2.09 1.99 0.91 1.00 3.20 3.16 2.96 5.75 2.78 5.78 16.82 7.73
f1 (ppm)

Sample Name	Pulse sequence **CARBON**	Temperature **25**	Study owner **cts**
Date collected	Solvent **cdcl3**	Spectrometer **inova500-inova500**	Operator **cts**

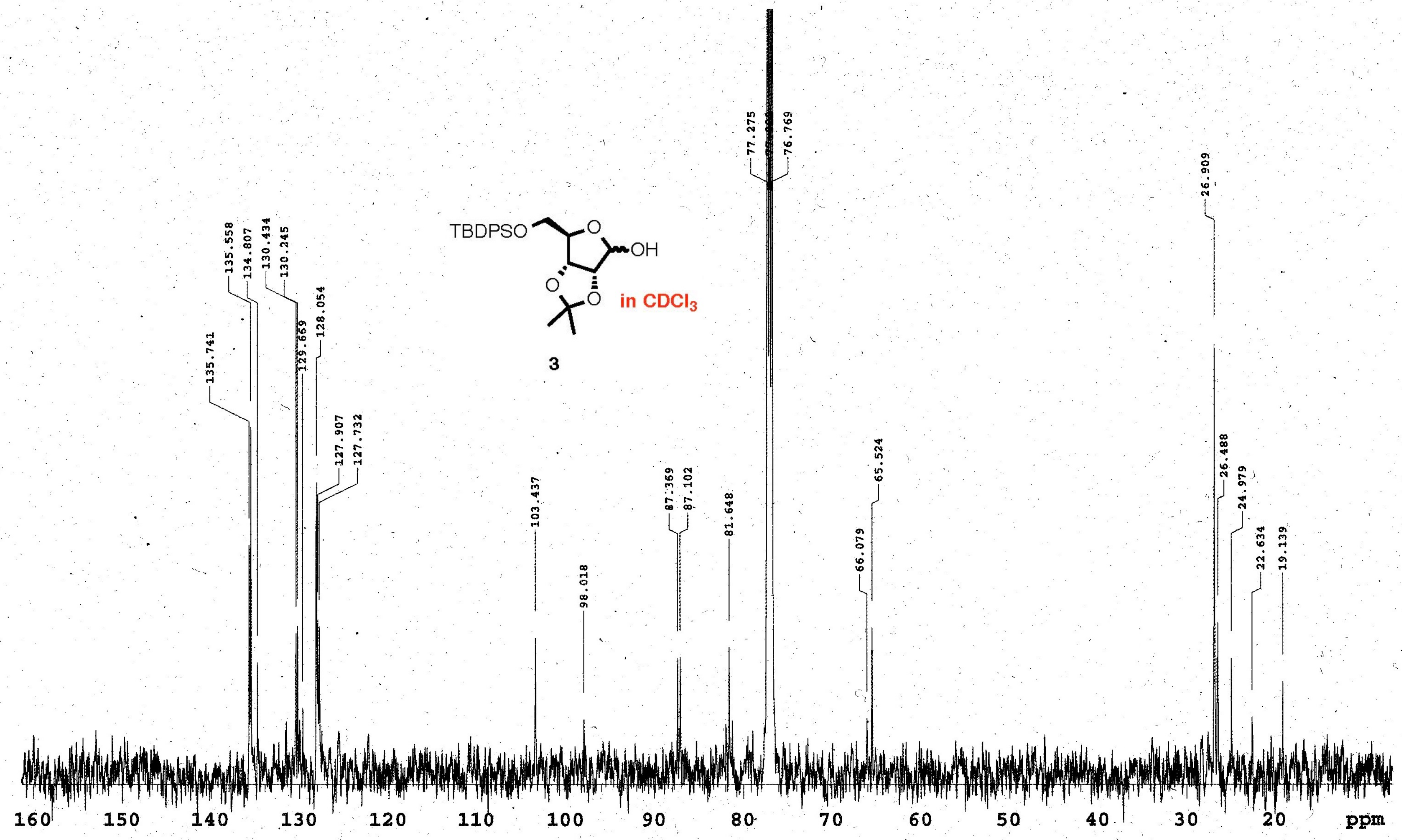

Data file /home/cts/vnmrsys/data/180423_AM1_fr9_20180423_01/CARBON_01

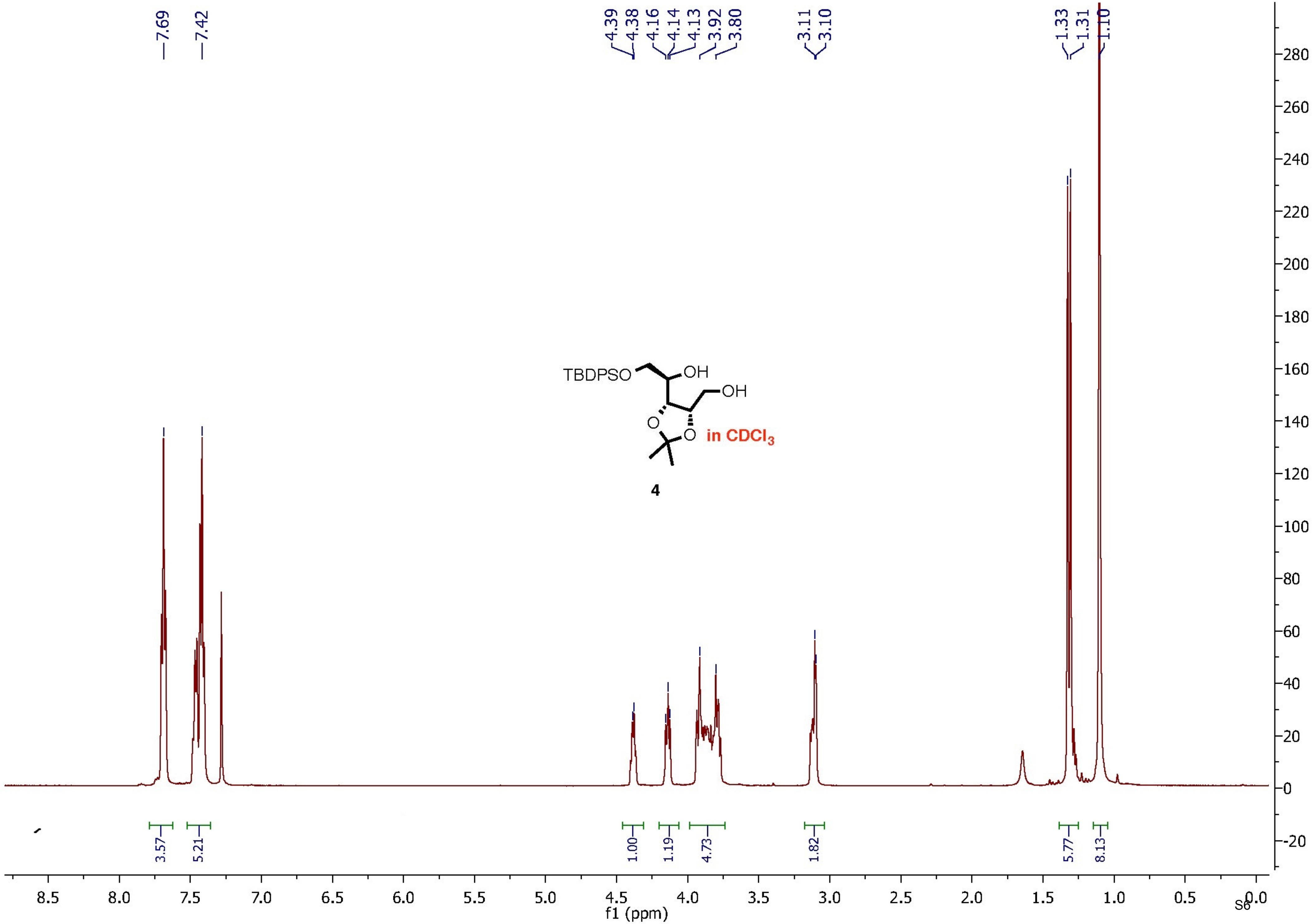

7.69
7.42
4.39
4.38
4.16
4.14
4.13
3.92
3.80
3.11
3.10
1.33
1.31
1.10
TBDPSO
OH
OH
O
O
in CDCl3
4
3.57
5.21
1.00
1.19
4.73
1.82
5.77
8.13
8.5 8.0 7.5 7.0 6.5 6.0 5.5 5.0 4.5 4.0 3.5 3.0 2.5 2.0 1.5 1.0 0.5 0.0
f1 (ppm)
280 260 240 220 200 180 160 140 120 100 80 60 40 20 0 -20

Sample Name
Date collected

Pulse sequence CARBON
Solvent cdcl3

Temperature 25
Spectrometer inova500-inova500

Study owner cts
Operator cts

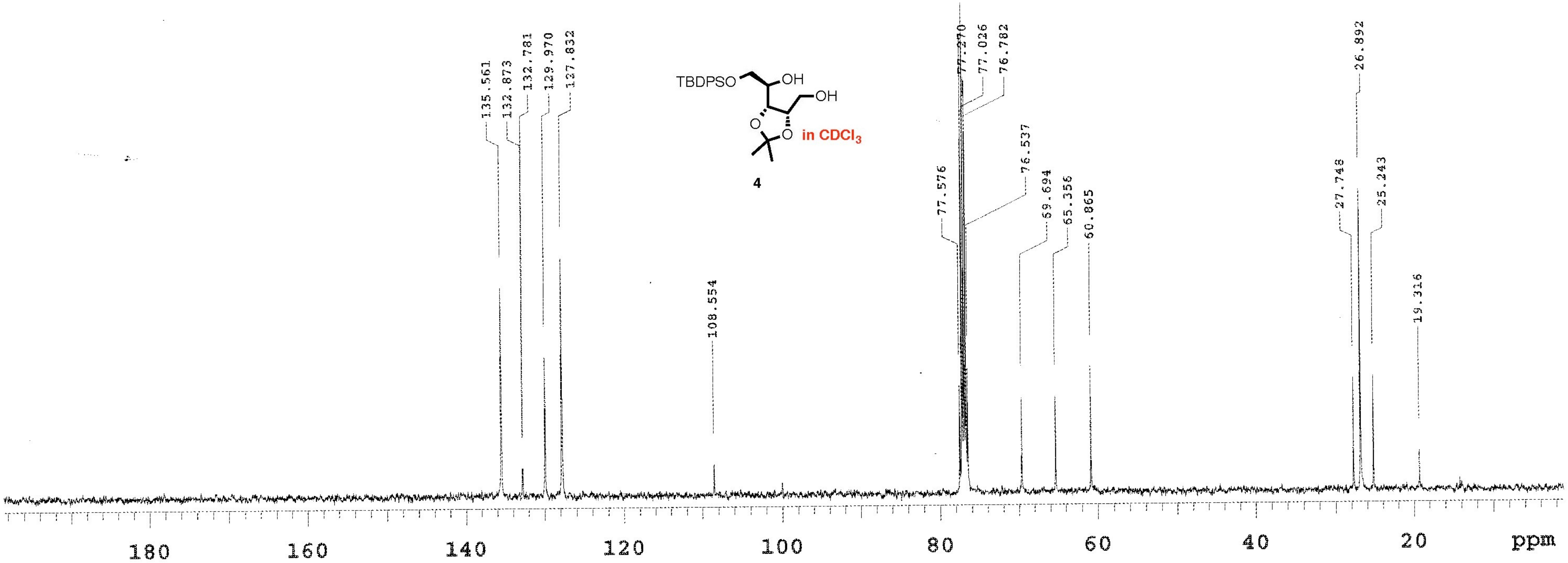

Plot date 2017-02-24

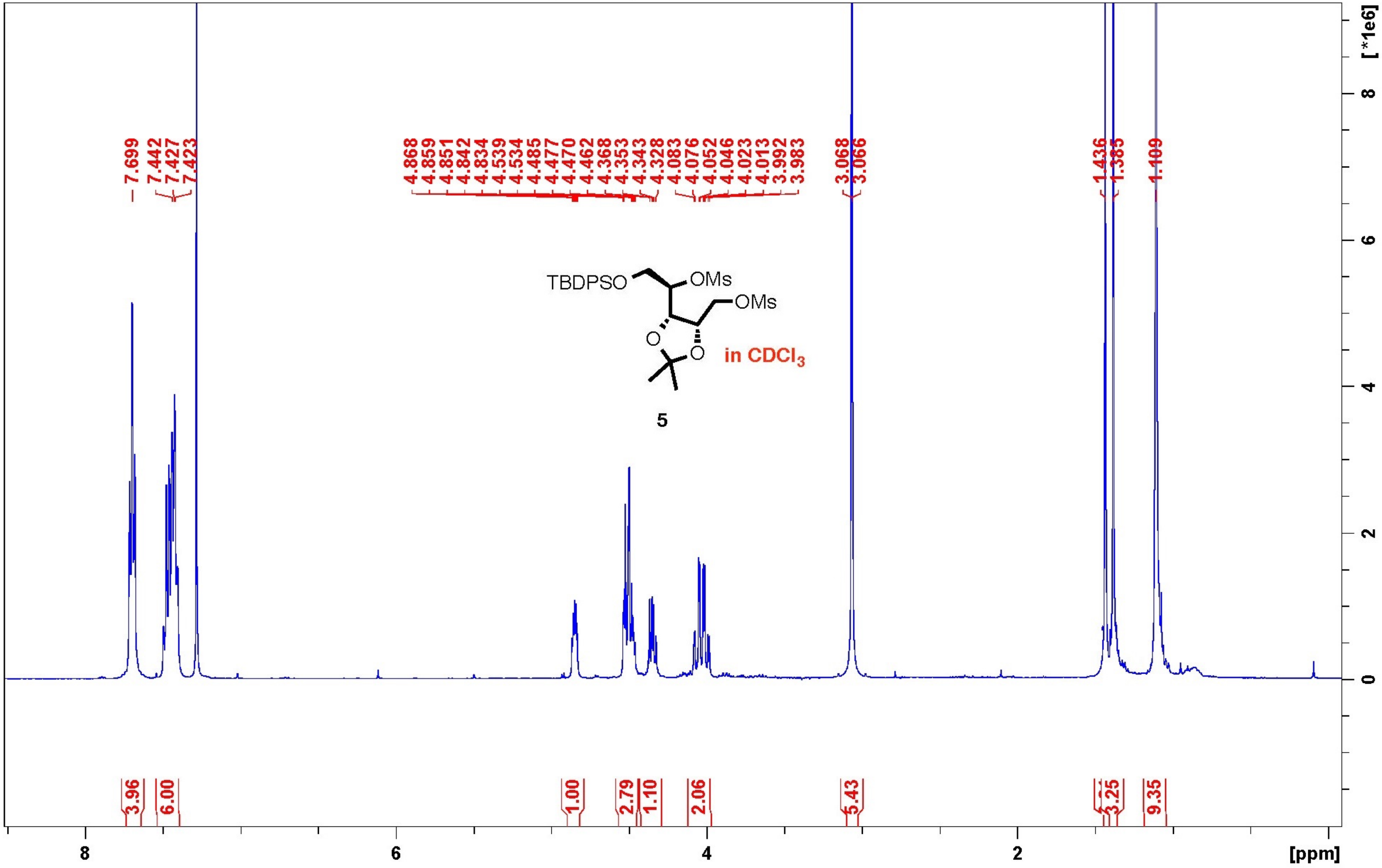

7.699
7.442
7.427
7.423
4.868
4.859
4.851
4.842
4.834
4.539
4.534
4.485
4.477
4.470
4.462
4.368
4.353
4.343
4.328
4.083
4.076
4.052
4.046
4.023
4.013
3.992
3.983
3.068
3.066
1.436
1.385
1.109
TBDPSO
OMs
OMs
O
O
in CDCl3
5
3.96
6.00
1.00
2.79
1.10
2.06
5.43
3.25
9.35
[ppm]
[*1e6]

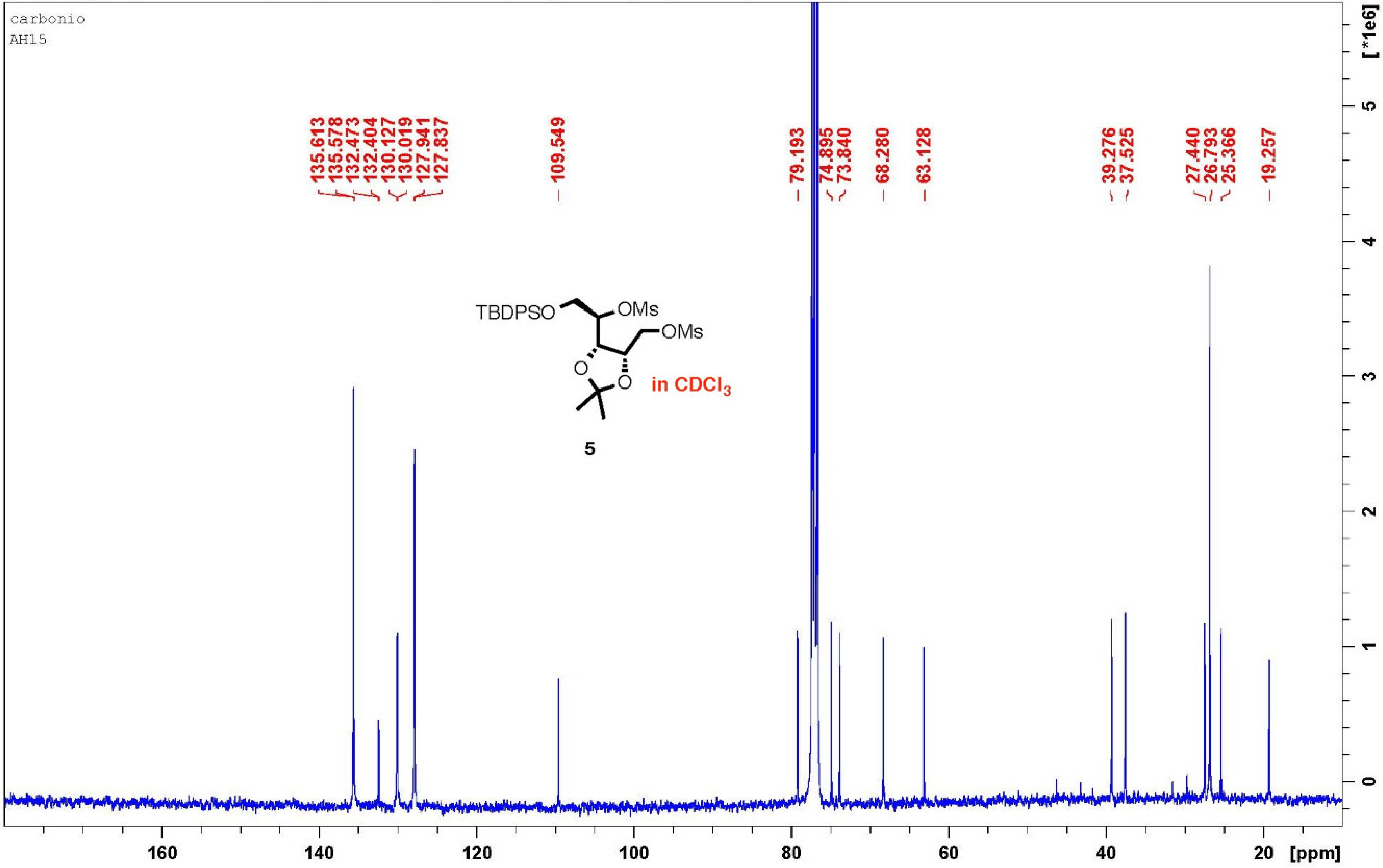
carbonio
AH15
135.613
135.578
132.473
132.404
130.127
130.019
127.941
127.837
109.549
79.193
74.895
73.840
68.280
63.128
39.276
37.525
27.440
26.793
25.366
19.257
TBDPSO
OMs
OMs
O
O
5
in CDCl3
[*1e6]
[ppm]

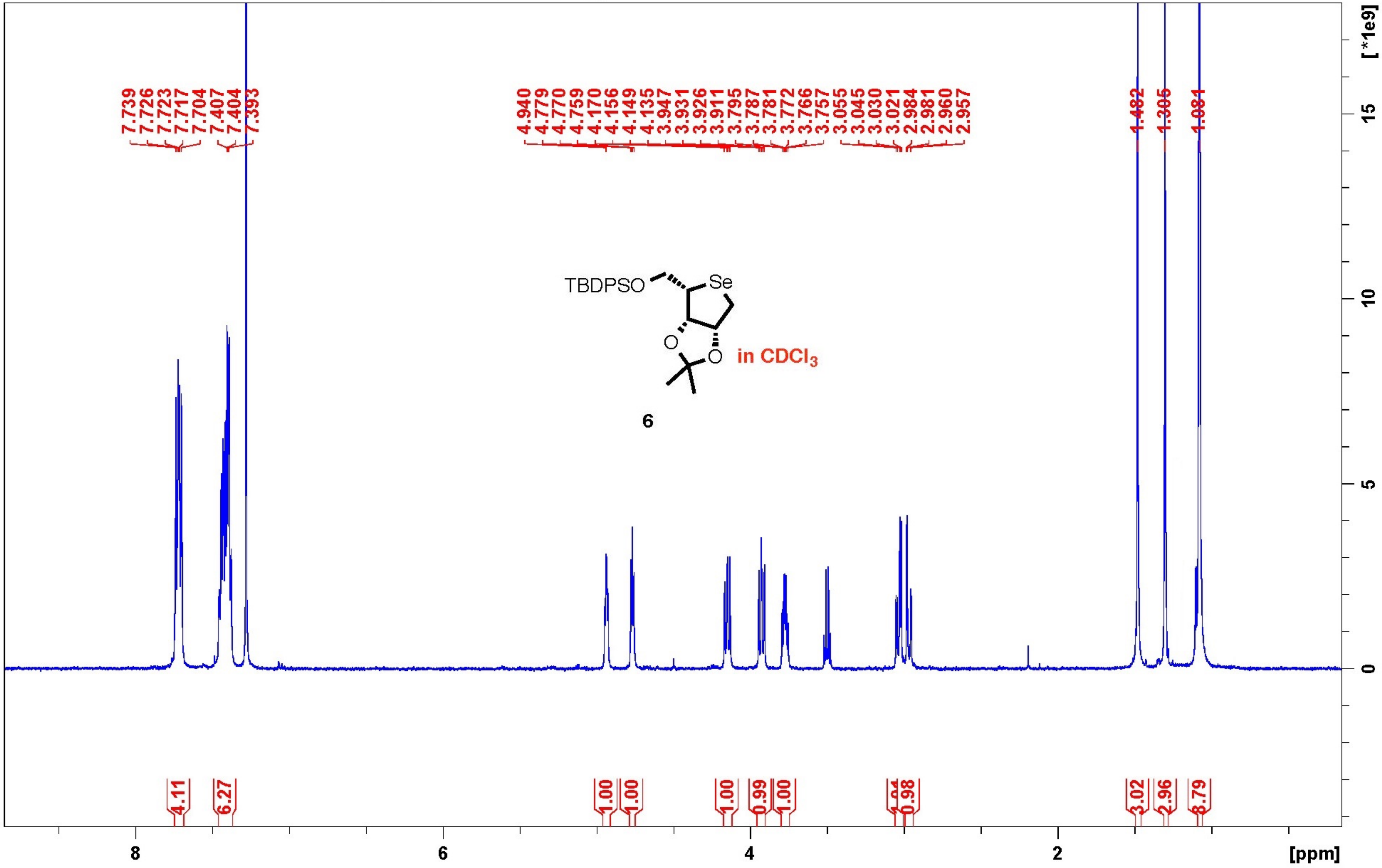

7.739
7.726
7.723
7.717
7.704
7.407
7.404
7.393
4.940
4.779
4.770
4.759
4.170
4.156
4.149
4.135
3.947
3.931
3.926
3.911
3.795
3.787
3.781
3.772
3.766
3.757
3.055
3.045
3.030
3.021
2.984
2.981
2.960
2.957
1.482
1.305
1.084
[*1e9]
15
10
5
0
TBDPSO
Se
O
O
in CDCl3
6
4.11
6.27
1.00
1.00
1.00
0.99
1.00
0.98
3.02
2.96
8.79
8
6
4
2
[ppm]

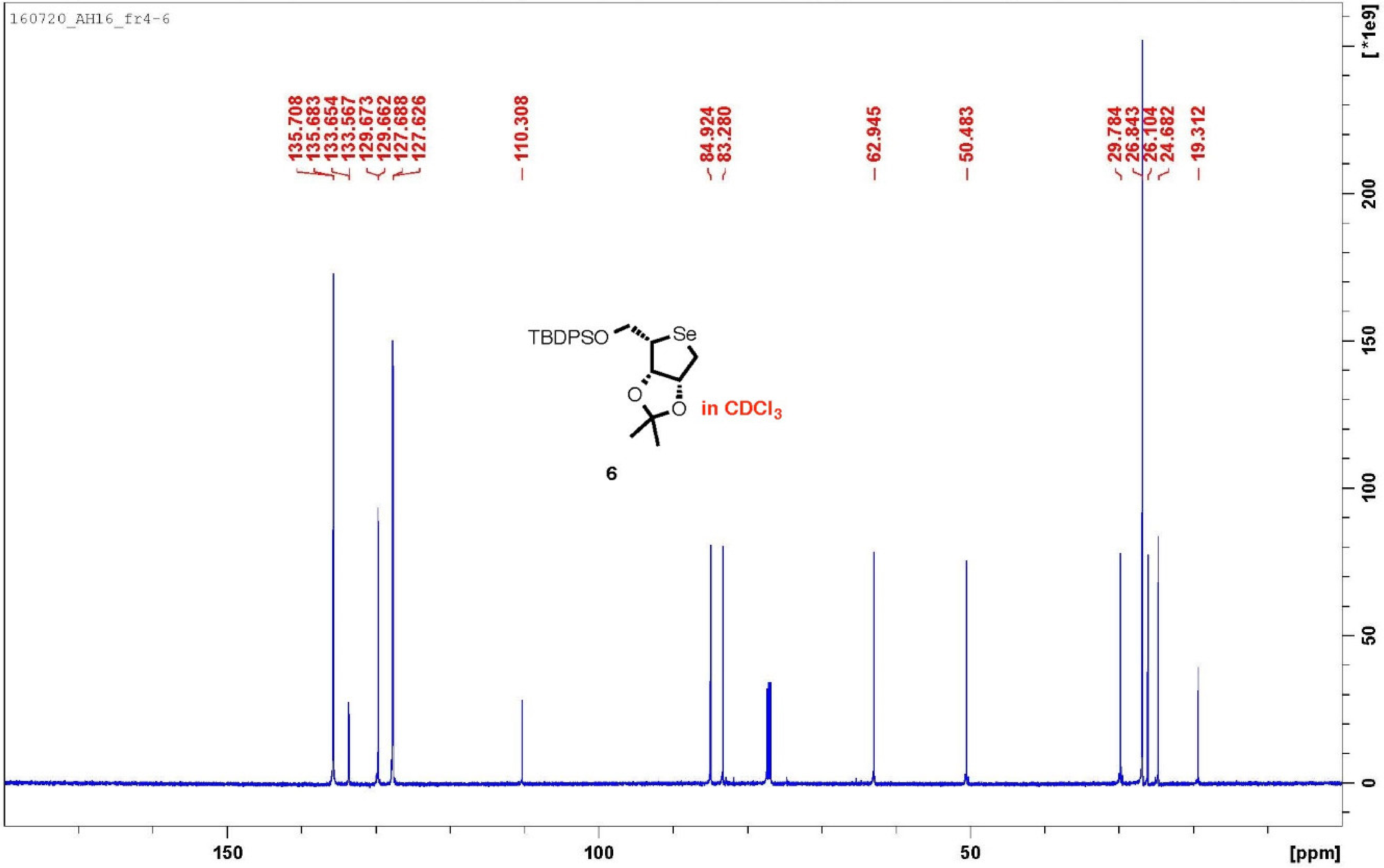

160720_AH16_fr4-6
135.708
135.683
133.654
133.567
129.673
129.662
127.688
127.626
110.308
84.924
83.280
62.945
50.483
29.784
26.843
26.104
24.682
19.312
TBDPSO
Se
O
O
in $CDCl_3$
6
[*1e9]
200
150
100
50
0
150
100
50
[ppm]

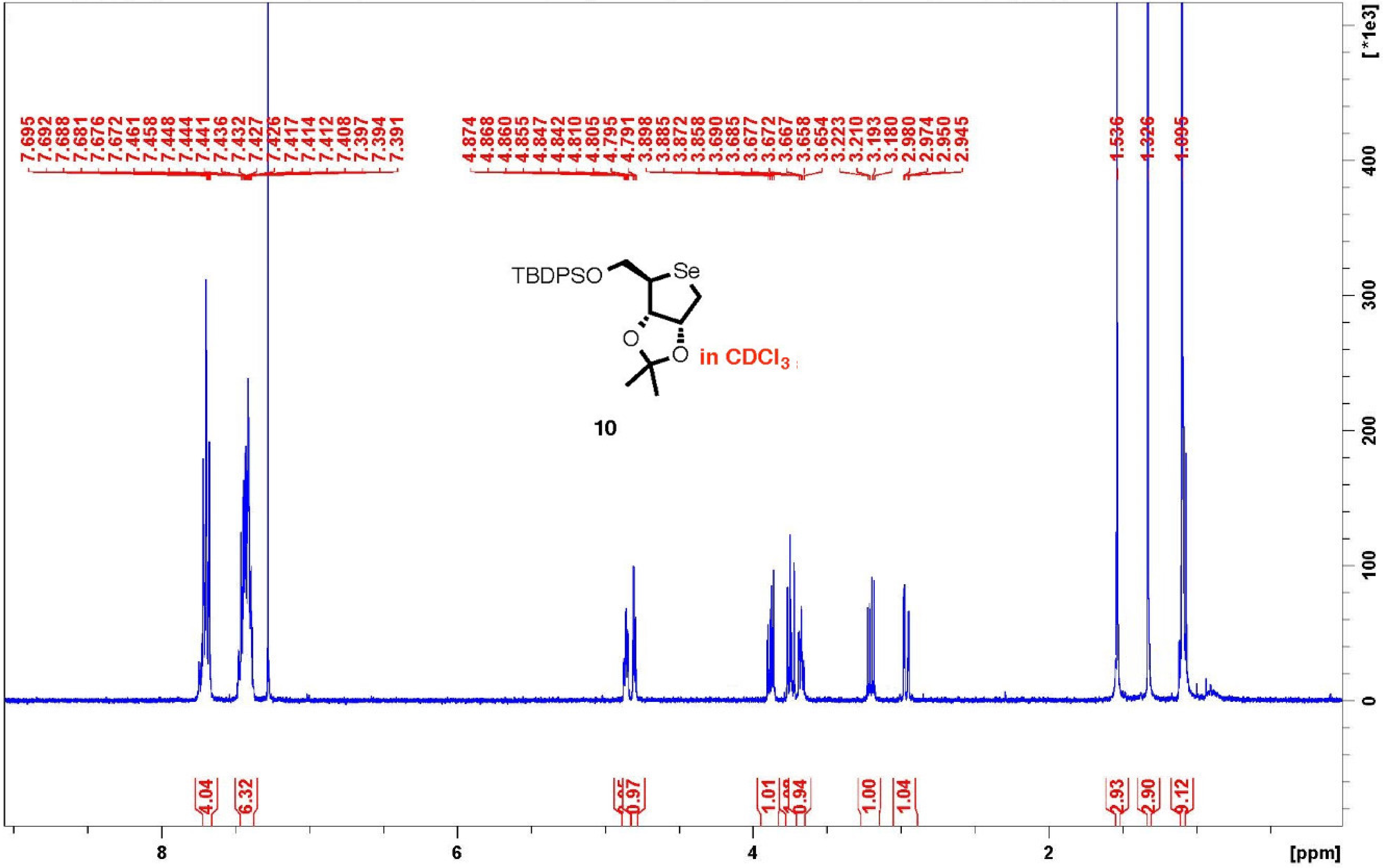
TBDPSO
Se
in CDCl3
10
7.695 7.692 7.688 7.681 7.676 7.672 7.461 7.458 7.448 7.444 7.441 7.436 7.432 7.427 7.426 7.417 7.414 7.412 7.408 7.397 7.394 7.391
4.874 4.868 4.860 4.855 4.847 4.842 4.810 4.805 4.795 4.791 3.898 3.885 3.872 3.858 3.690 3.685 3.677 3.672 3.667 3.658 3.654 3.223 3.210 3.193 3.180 2.980 2.974 2.950 2.945
1.536
1.326
1.095
4.04
6.32
0.97
1.01
0.94
1.00
1.04
2.93
2.90
9.12
[*1e3]
[ppm]

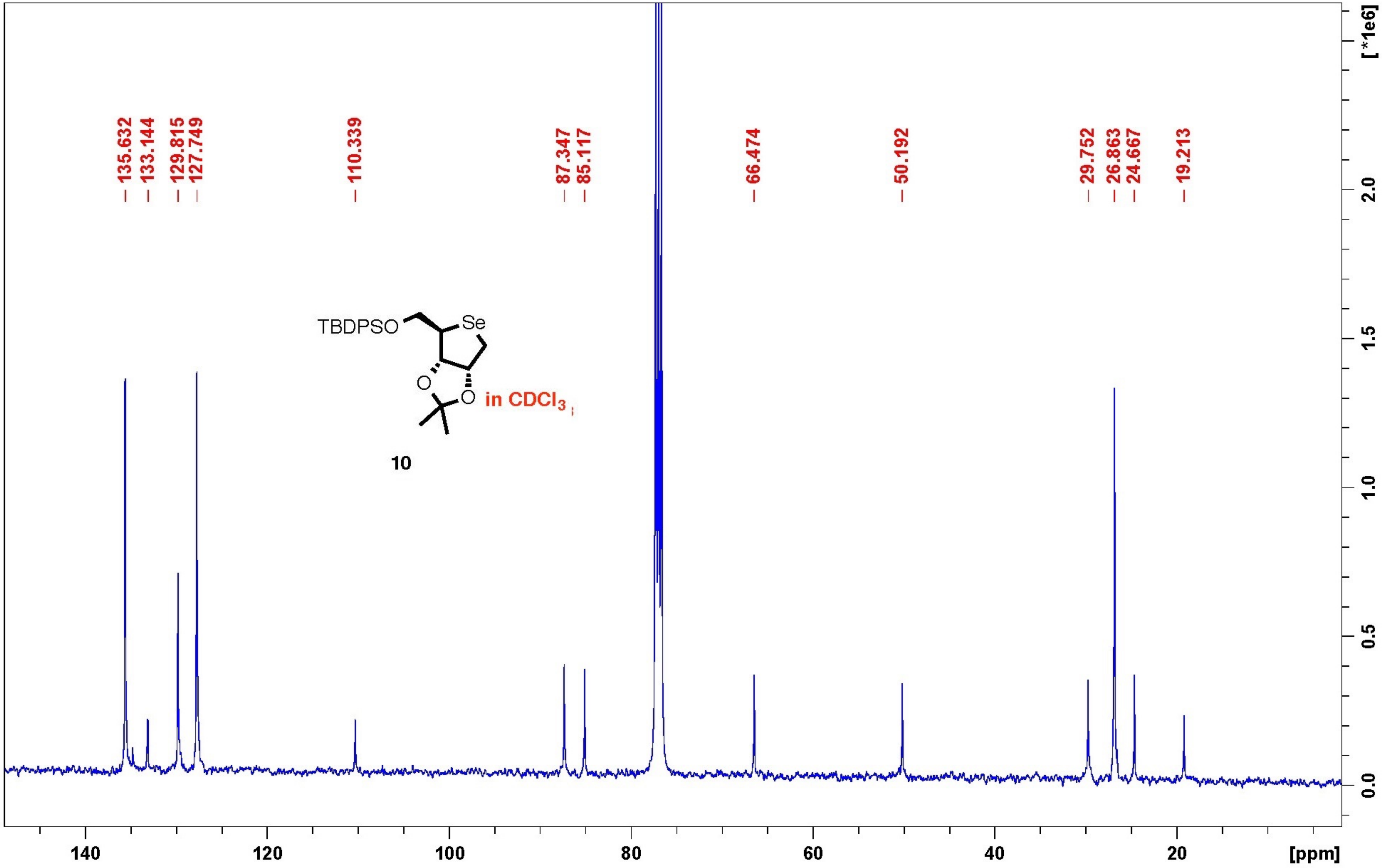

135.632
133.144
129.815
127.749
110.339
87.347
85.117
66.474
50.192
29.752
26.863
24.667
19.213
TBDPSO
Se
O
O
in CDCl3
10
[*1e6]
2.0
1.5
1.0
0.5
0.0
140
120
100
80
60
40
20
[ppm]

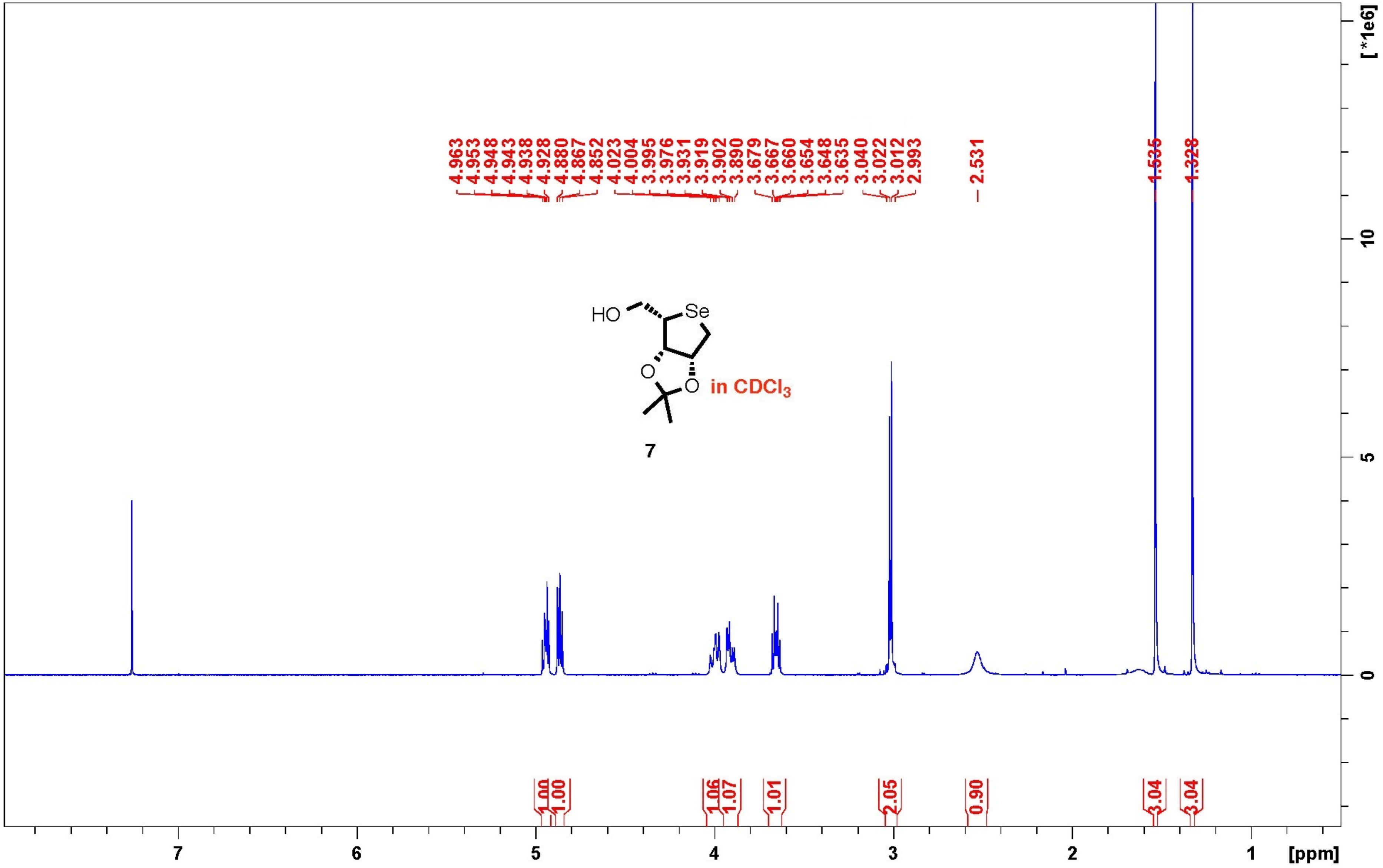

4.963
4.953
4.948
4.943
4.938
4.928
4.880
4.867
4.852
4.023
4.004
3.995
3.976
3.931
3.919
3.902
3.890
3.679
3.667
3.660
3.654
3.648
3.635
3.040
3.022
3.012
2.993
2.531
1.535
1.328
HO
Se
O
O
in $CDCl_3$
7
1.00
1.00
1.06
1.07
1.01
2.05
0.90
3.04
3.04
7
6
5
4
3
2
1
[ppm]
[*1e6]
10
5
0

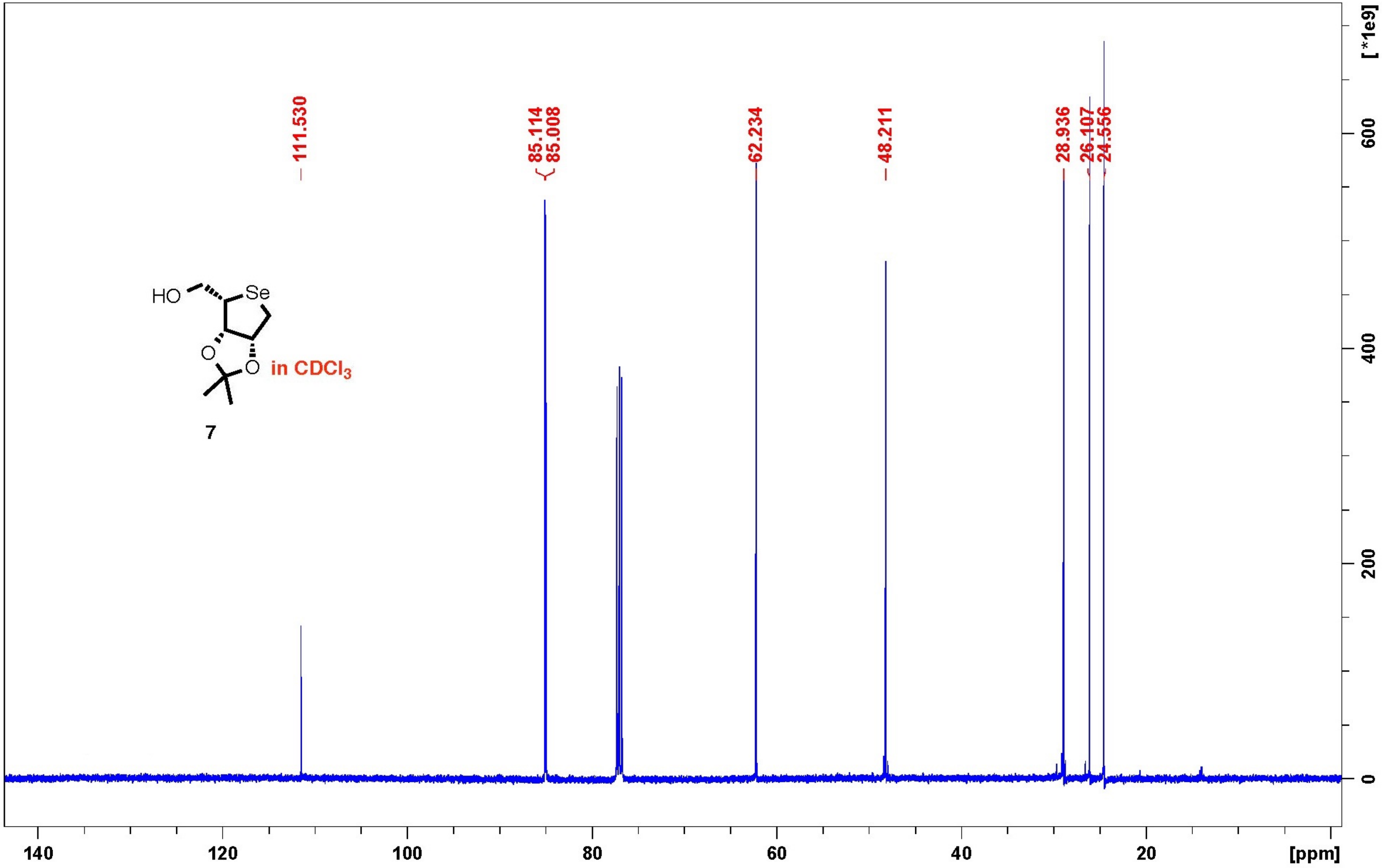
111.530
85.114
85.008
62.234
48.211
28.936
26.107
24.556
[*1e9]
600
400
200
0
HO
Se
O
O
in CDCl3
7
140
120
100
80
60
40
20
[ppm]

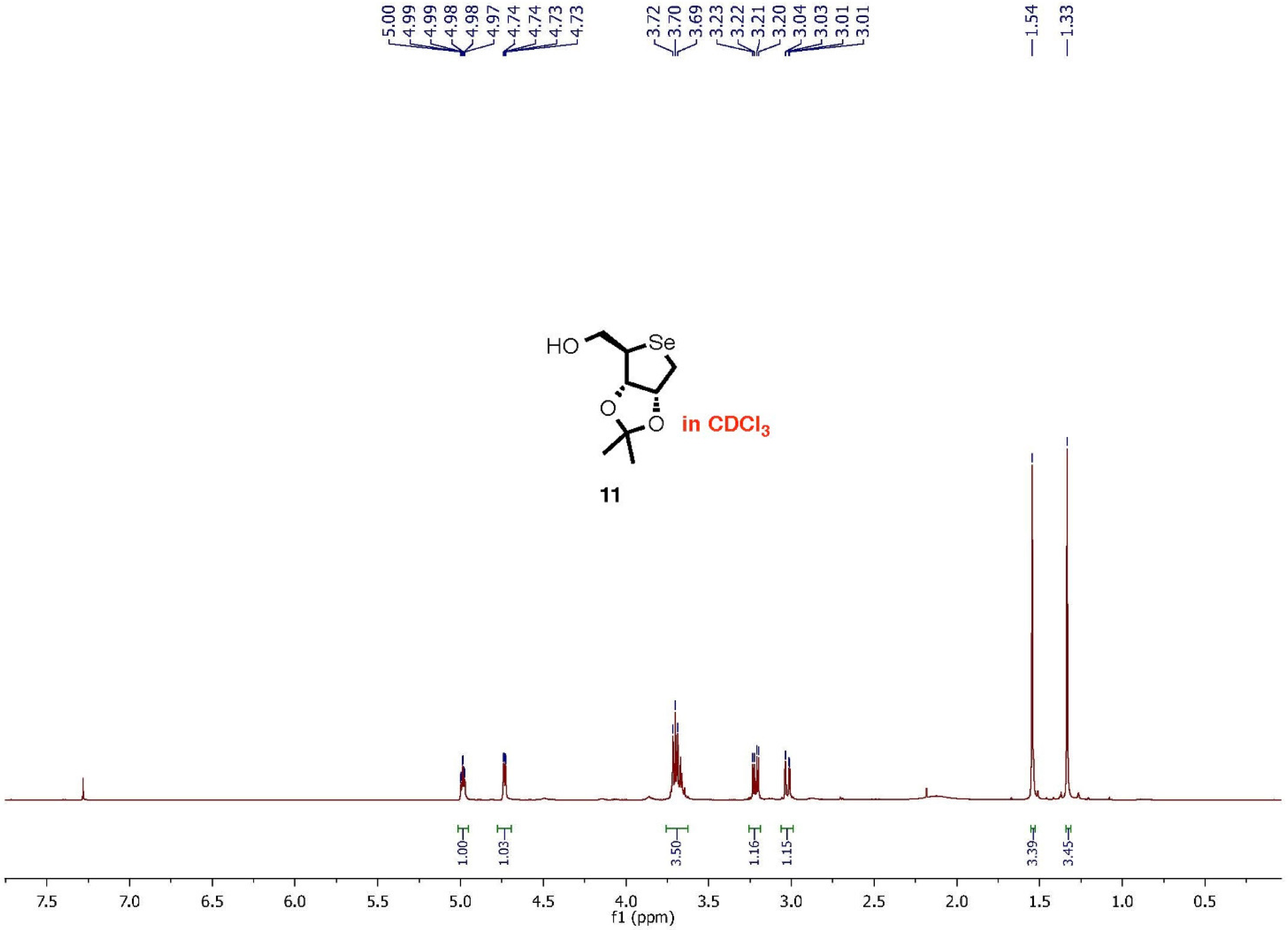
5.00 4.99 4.99 4.98 4.98 4.97 4.74 4.74 4.73 4.73
3.72 3.70 3.69 3.23 3.22 3.21 3.20 3.04 3.03 3.01 3.01
1.54
1.33
HO
Se
O
O
in $CDCl_3$
11
1.00
1.03
3.50
1.16
1.15
3.39
3.45
7.5 7.0 6.5 6.0 5.5 5.0 4.5 4.0 3.5 3.0 2.5 2.0 1.5 1.0 0.5
f1 (ppm)

Sample Name
Date collected

Pulse sequence CARBON
Solvent cdcl3

Temperature 25
Spectrometer inova500-inova500

Study owner cts
Operator cts

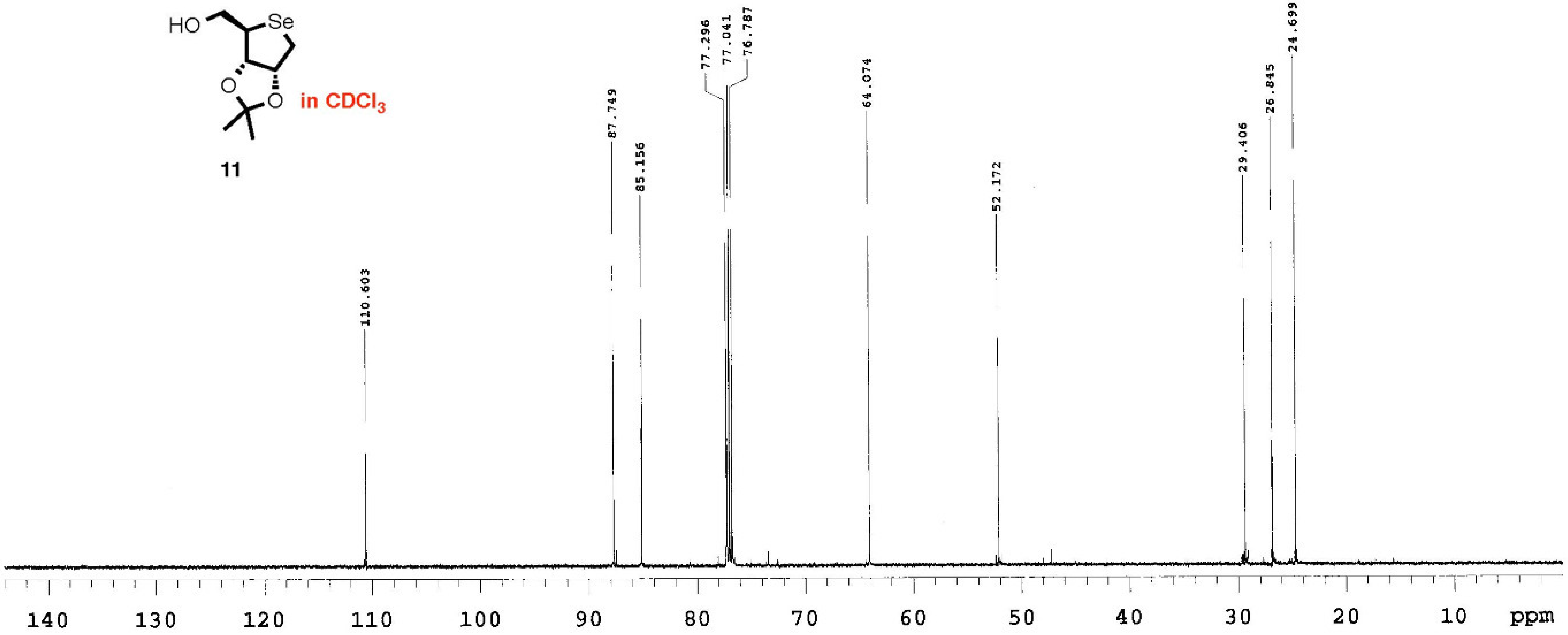

Data file [illegible]

Plot date [illegible]

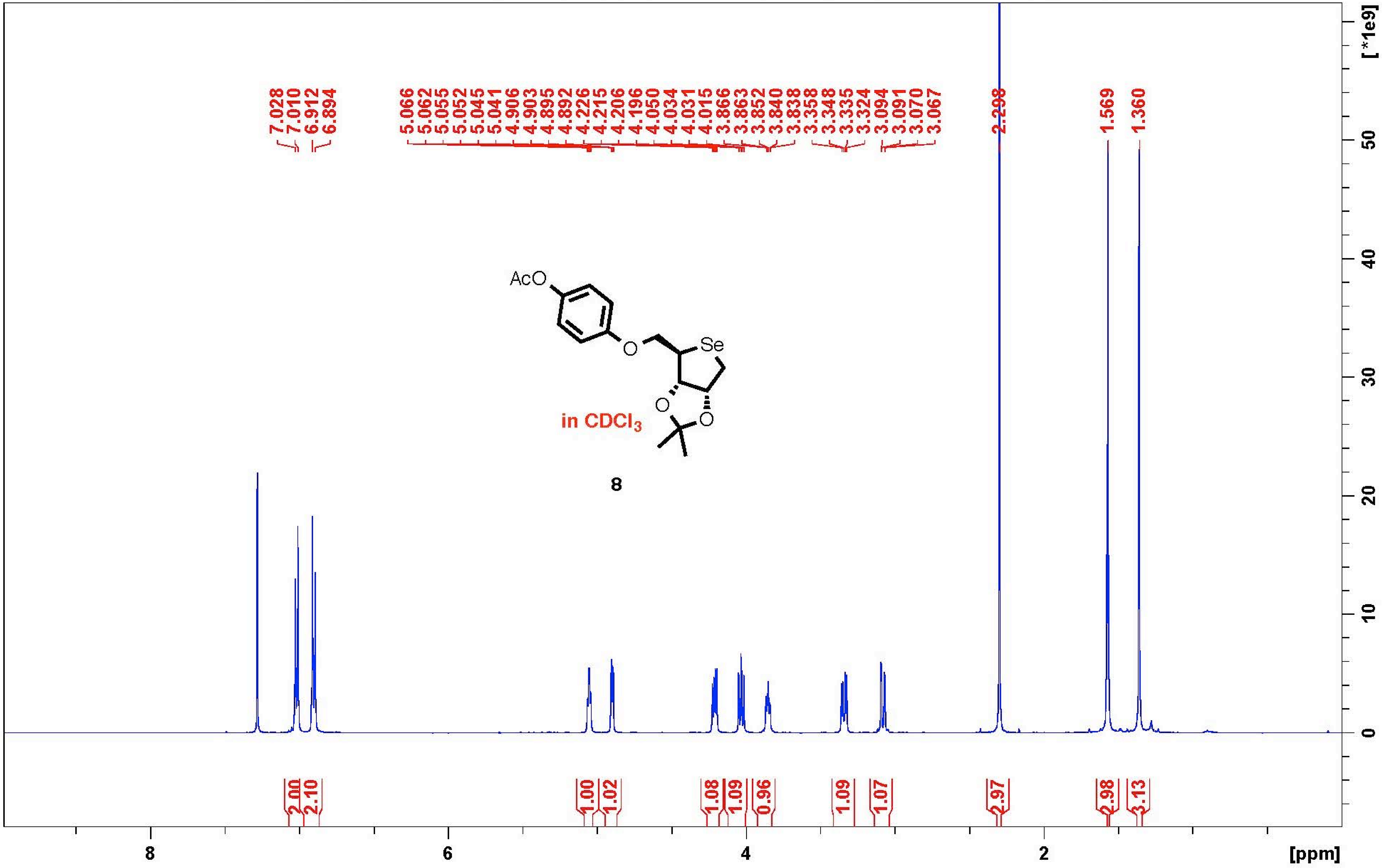
7.028
7.010
6.912
6.894
5.066
5.062
5.055
5.052
5.045
5.041
4.906
4.903
4.895
4.892
4.226
4.215
4.206
4.196
4.050
4.034
4.031
4.015
3.866
3.863
3.852
3.840
3.838
3.358
3.348
3.335
3.324
3.094
3.091
3.070
3.067
2.298
1.569
1.360
AcO
Se
O
in $CDCl_3$
2.00
2.10
1.00
1.02
1.08
1.09
0.96
1.09
1.07
2.97
2.98
3.13
[ppm]
[*1e9]

8

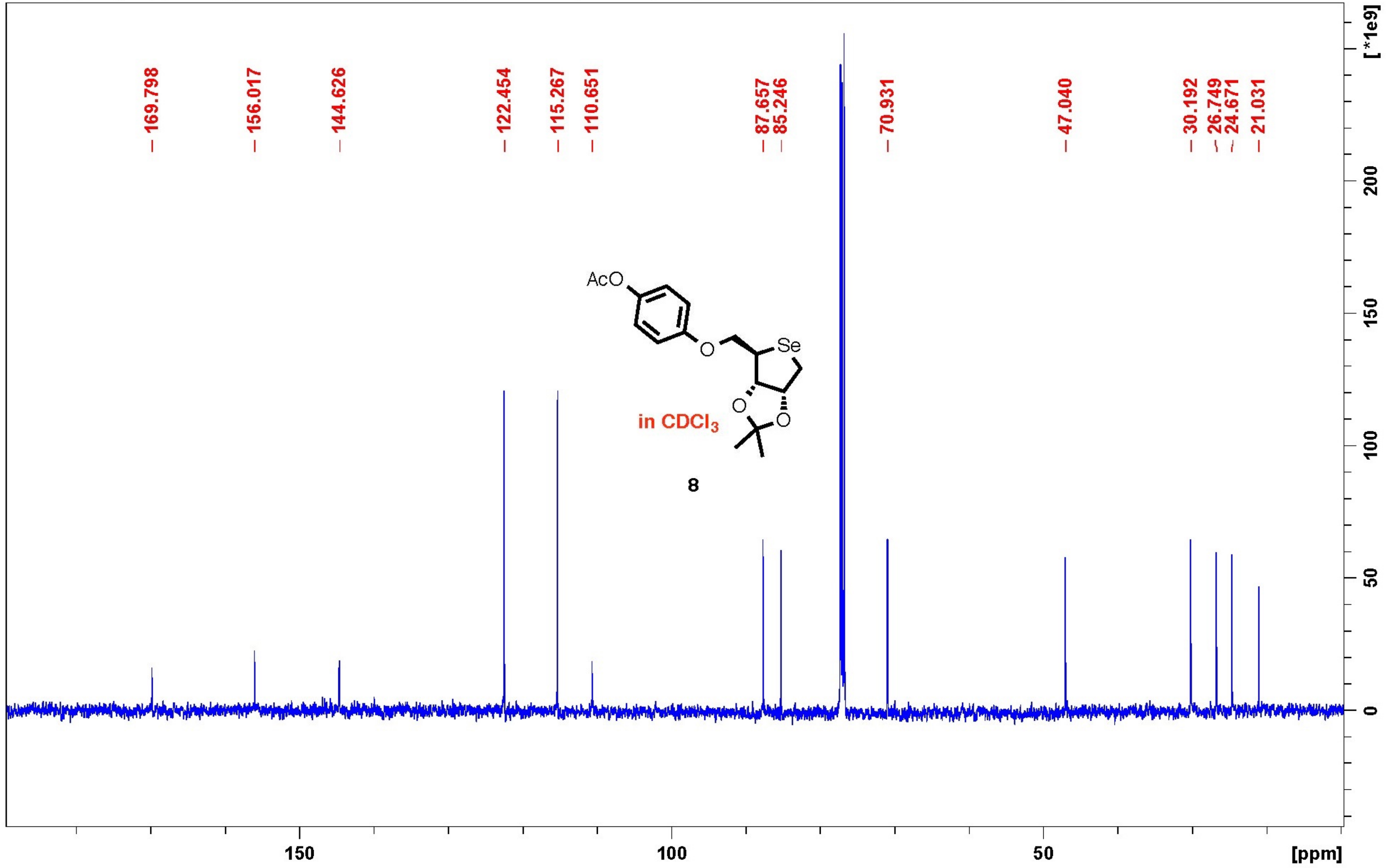
169.798
156.017
144.626
122.454
115.267
110.651
87.657
85.246
70.931
47.040
30.192
26.749
24.671
21.031
AcO
O
Se
O
O
in $CDCl_3$
8
150
100
50
[ppm]
[*1e9]
200
150
100
50
0

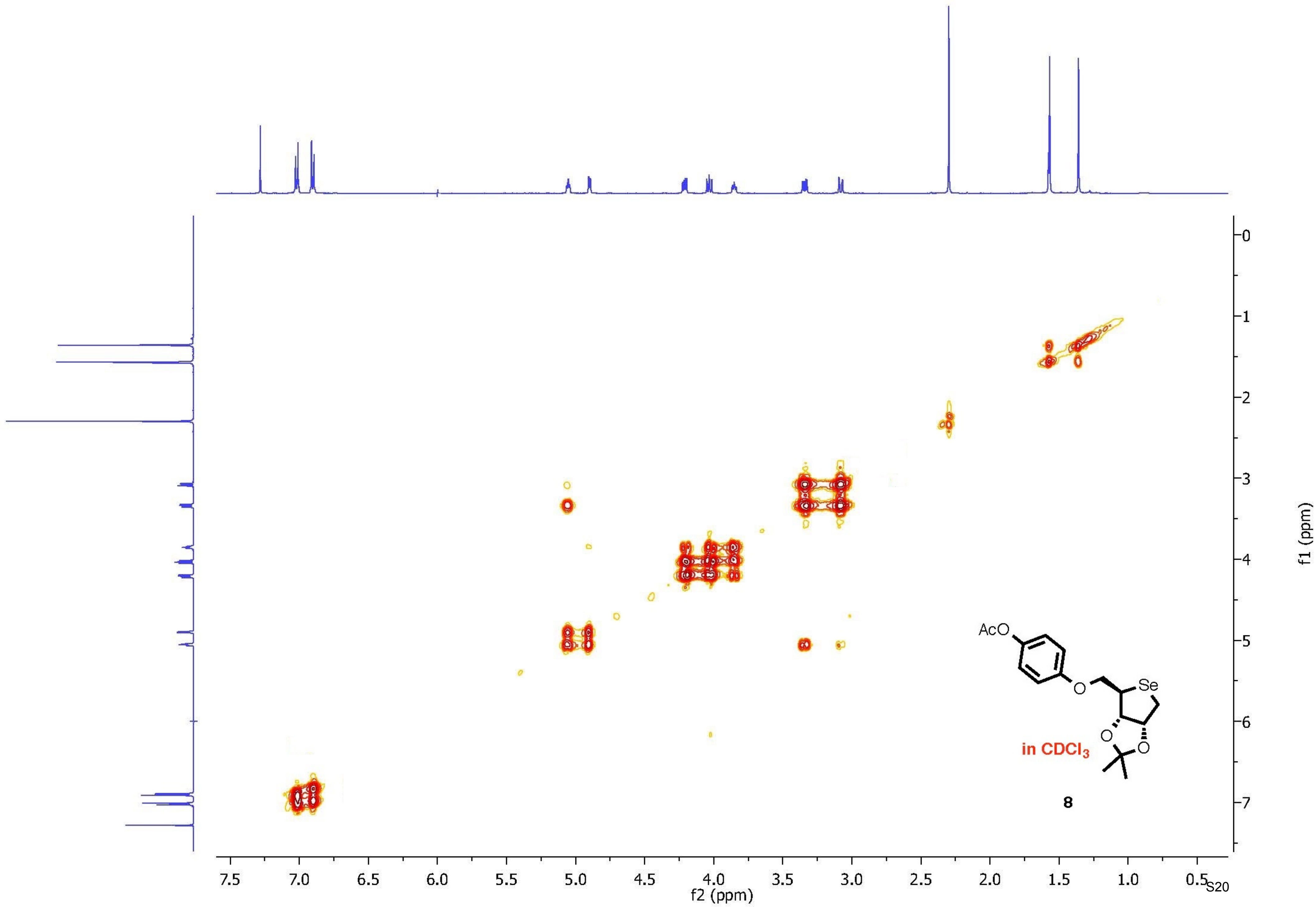

8

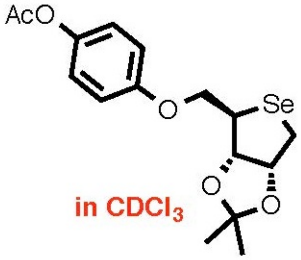

8

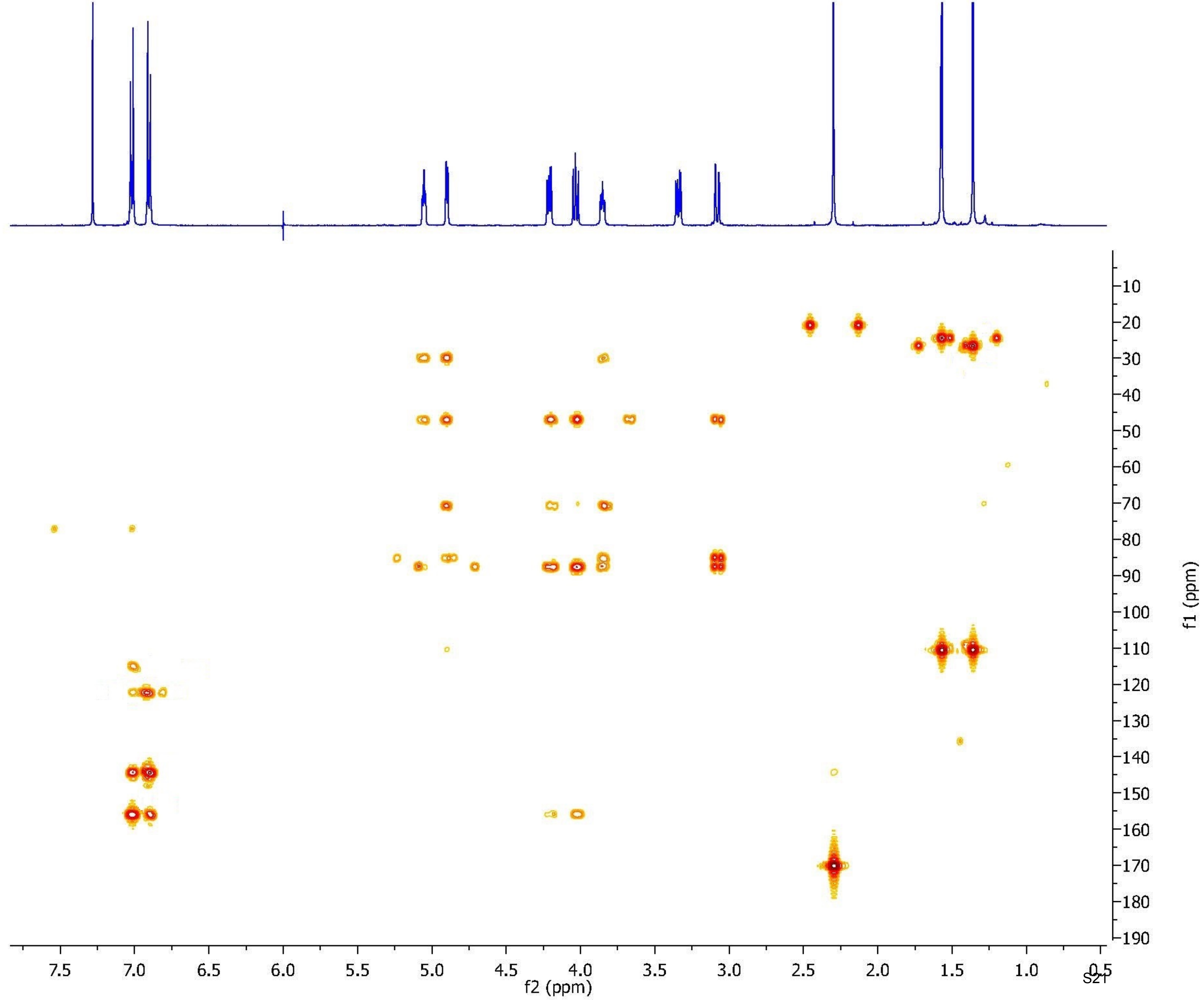

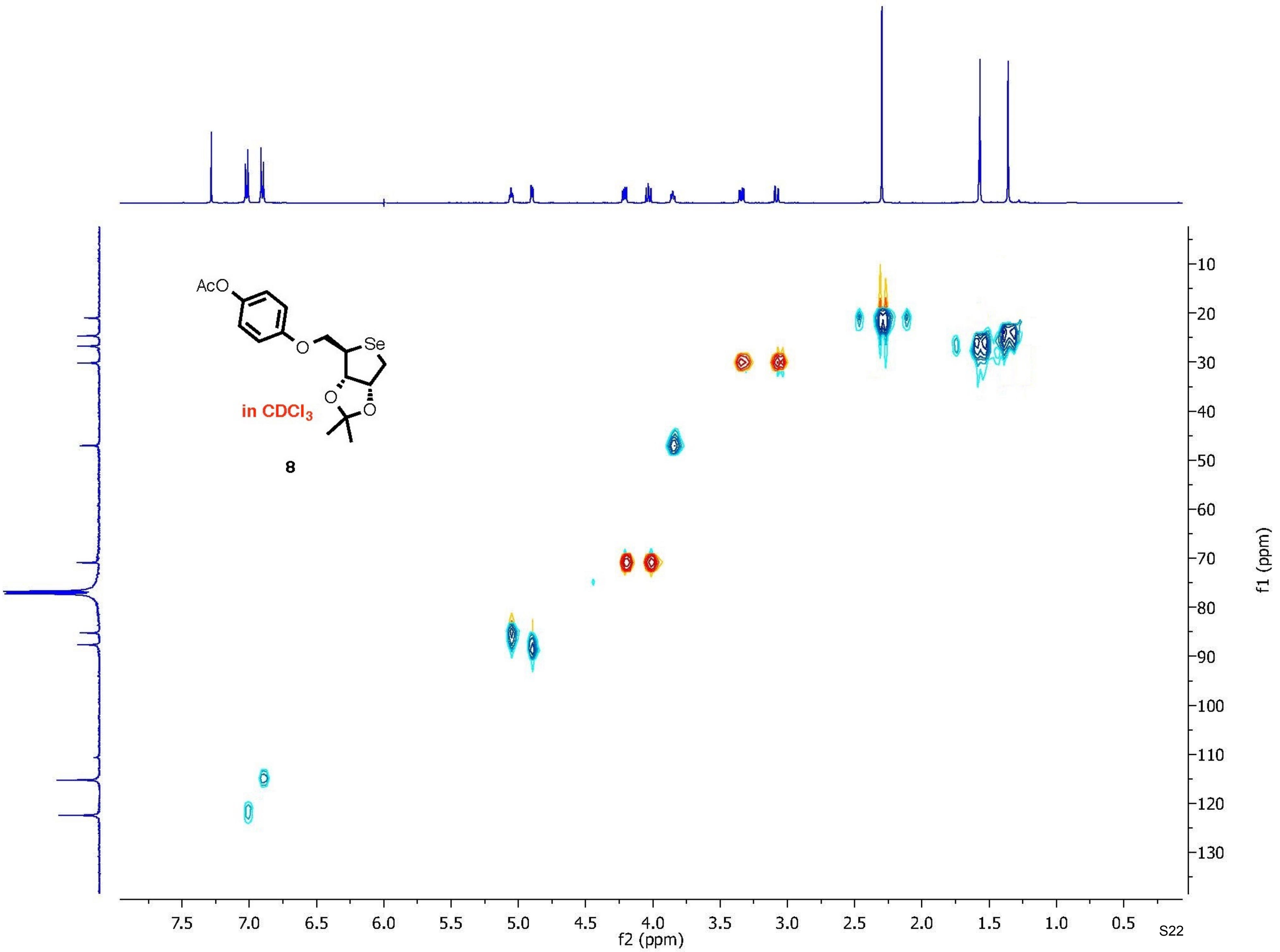

AcO
Se
O
in CDCl3
8
f1 (ppm)
f2 (ppm)

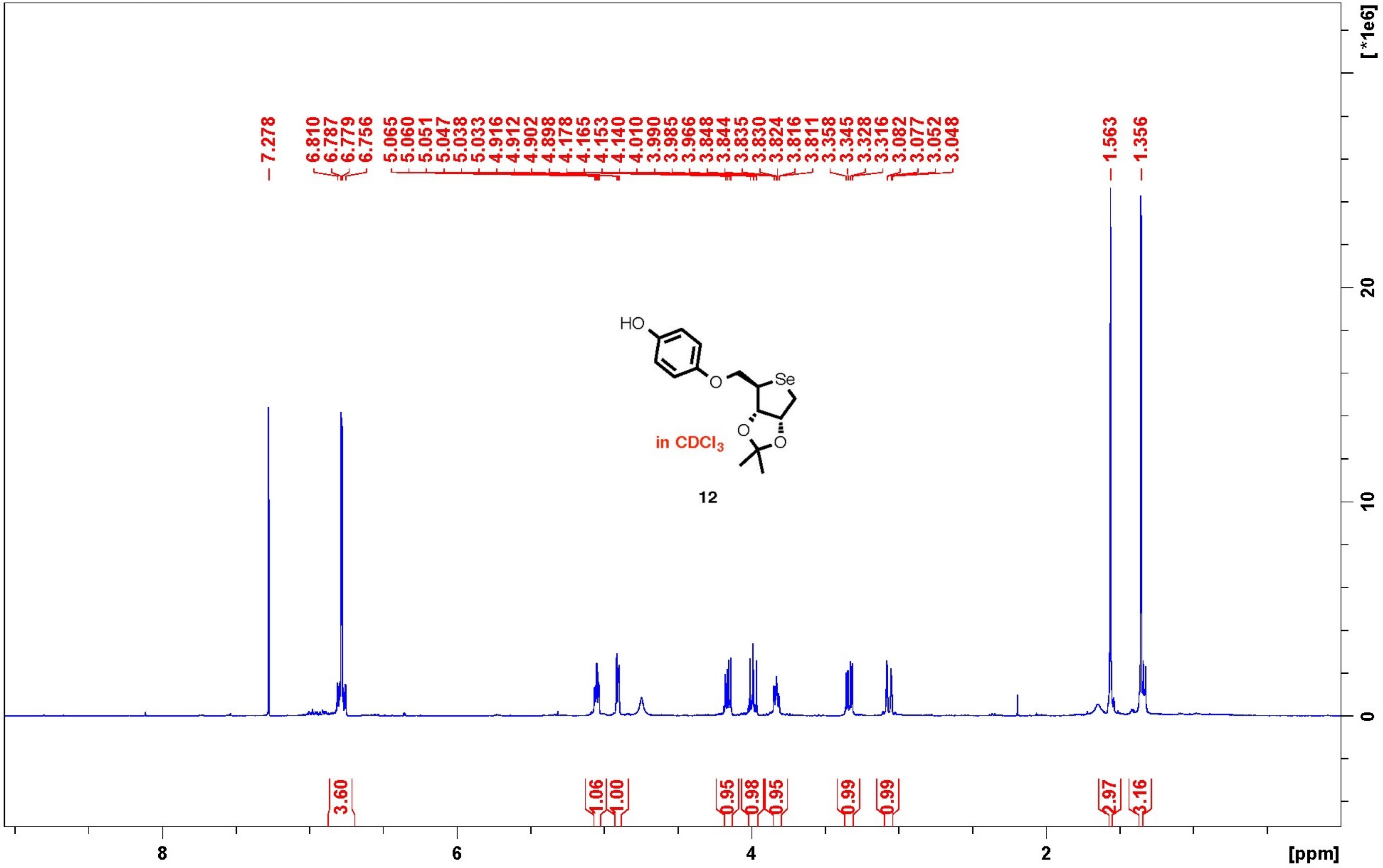

7.278
6.810
6.787
6.779
6.756
5.065
5.060
5.051
5.047
5.038
5.033
4.916
4.912
4.902
4.898
4.178
4.165
4.153
4.140
4.010
3.990
3.985
3.966
3.848
3.844
3.835
3.830
3.824
3.816
3.811
3.358
3.345
3.328
3.316
3.082
3.077
3.052
3.048
1.563
1.356
HO
O
Se
O
O
in CDCl3
12
3.60
1.06
1.00
0.95
0.98
0.95
0.99
0.99
2.97
3.16
8
6
4
2
[ppm]
[*1e6]
20
10
0

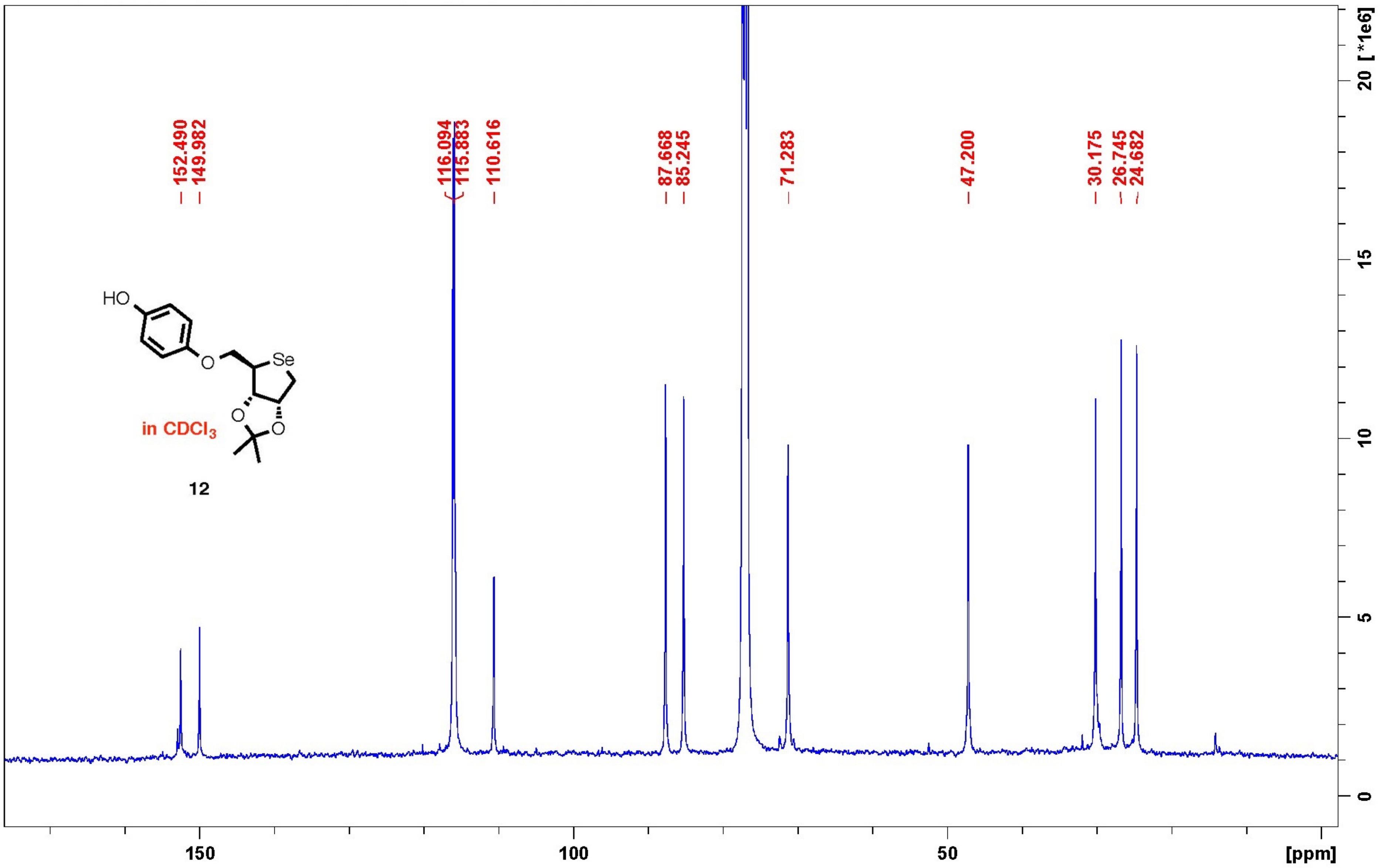
152.490
149.982
116.094
115.883
110.616
87.668
85.245
71.283
47.200
30.175
26.745
24.682
HO
O
Se
O
O
in CDCl3
12
20 [*1e6]
15
10
5
0
150
100
50
[ppm]

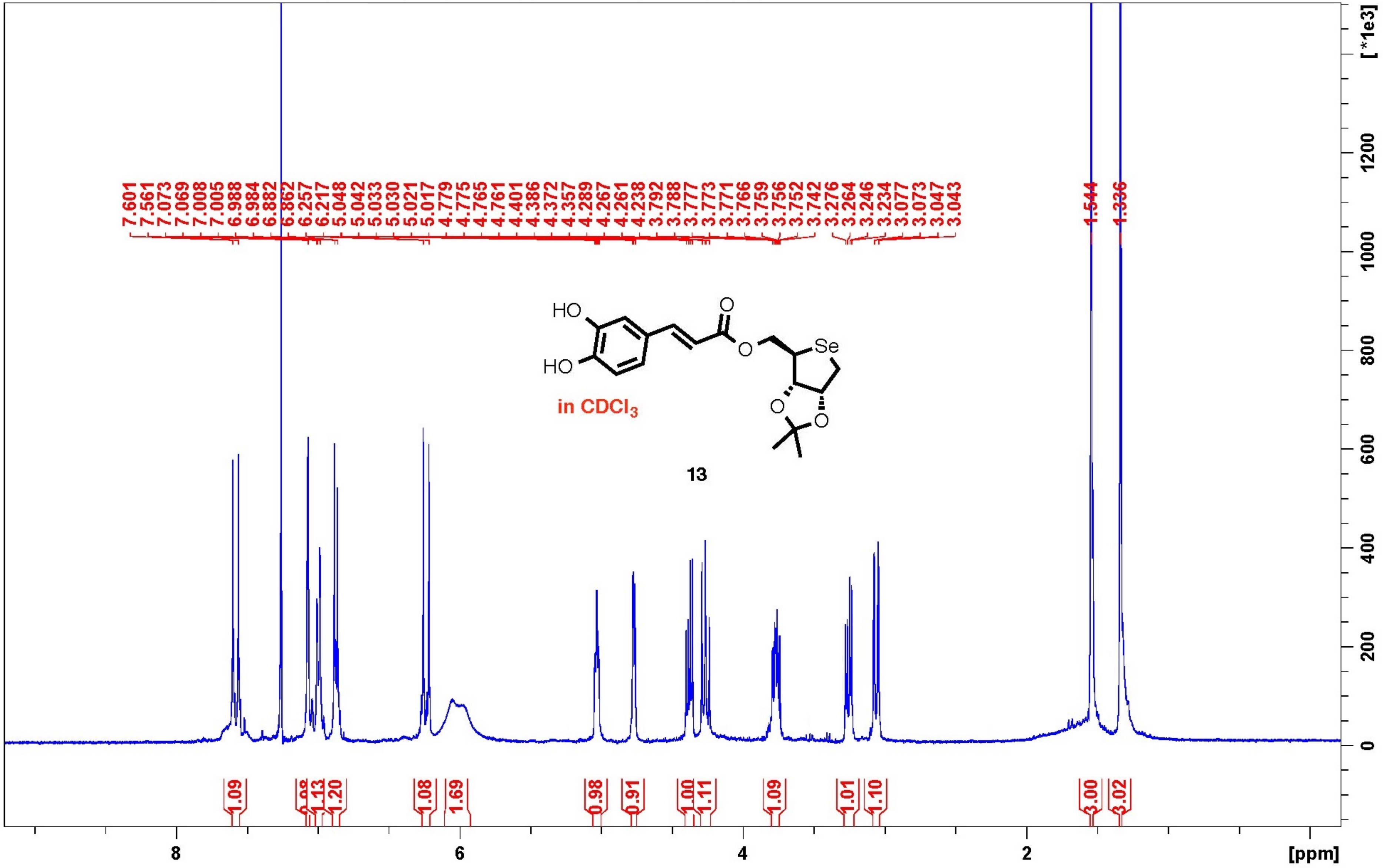

7.601
7.561
7.073
7.069
7.008
7.005
6.988
6.984
6.882
6.862
6.257
6.217
5.048
5.042
5.033
5.030
5.021
5.017
4.779
4.775
4.765
4.761
4.401
4.386
4.372
4.357
4.289
4.267
4.261
4.238
3.792
3.788
3.777
3.773
3.771
3.766
3.759
3.756
3.752
3.742
3.276
3.264
3.246
3.234
3.077
3.073
3.047
3.043
1.544
1.336
HO
HO
O
O
Se
O
O
in CDCl3
13
1.09
1.13
1.20
1.08
1.69
0.98
0.91
1.00
1.11
1.09
1.01
1.10
3.00
3.02
8
6
4
2
[ppm]
[*1e3]
1200
1000
800
600
400
200
0

Sample Name
Date collected

Pulse sequence **CARBON**
Solvent **cdcl3**

Temperature **28**
Spectrometer **inova500-inova500**

Study owner **cts**
Operator **cts**

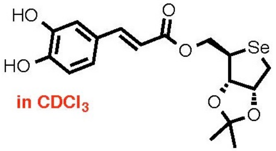

in $CDCl_3$

13

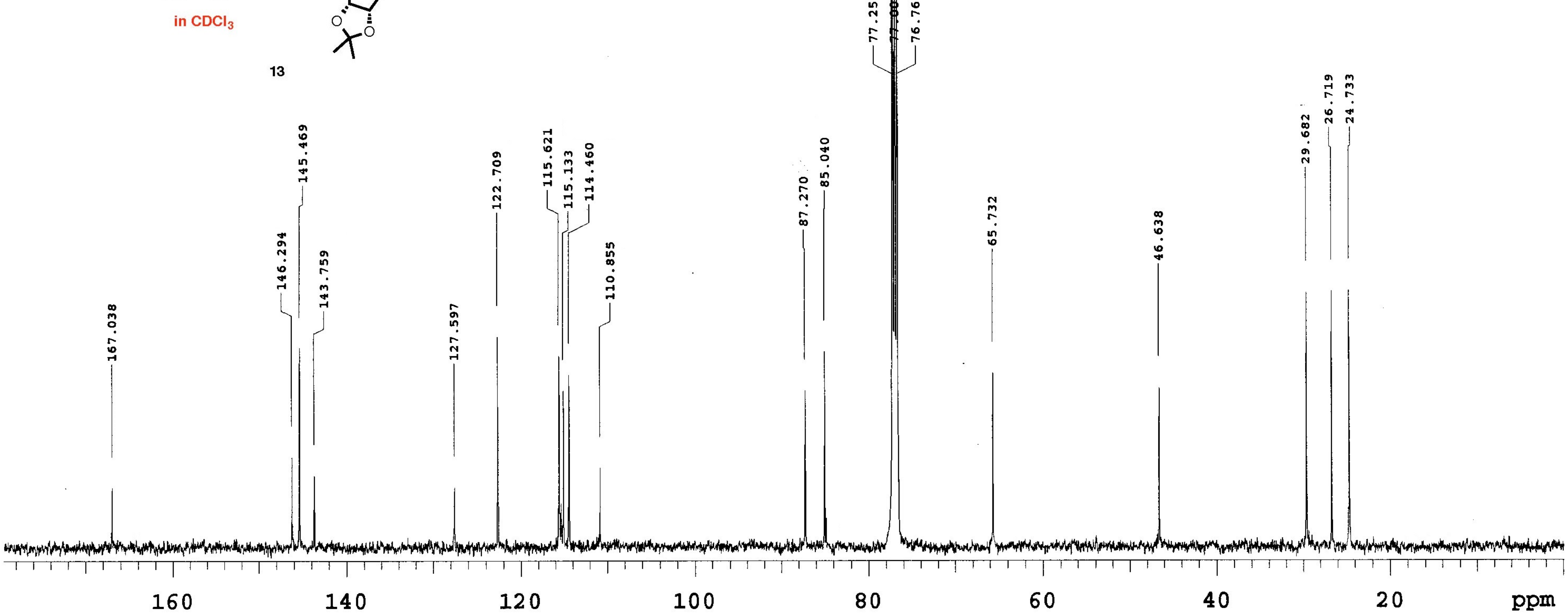

Data file exp

Plot date 2019-09-09

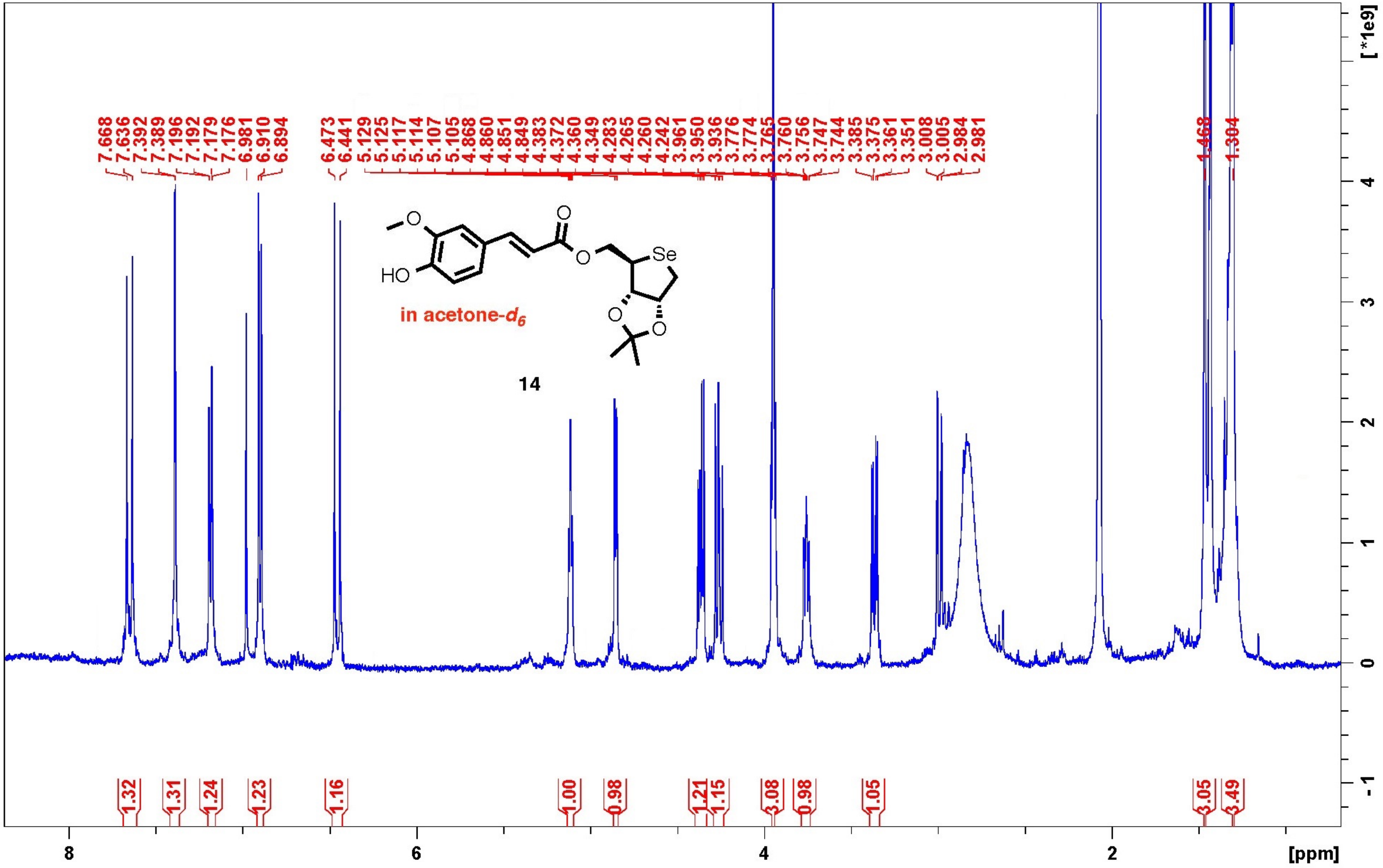

7.668
7.636
7.392
7.389
7.196
7.192
7.179
7.176
6.981
6.910
6.894
6.473
6.441
5.129
5.125
5.117
5.114
5.107
5.105
4.868
4.860
4.851
4.849
4.383
4.372
4.360
4.349
4.283
4.265
4.260
4.242
3.961
3.950
3.936
3.774
3.765
3.760
3.756
3.747
3.744
3.385
3.375
3.361
3.351
3.008
3.005
2.984
2.981
1.468
1.304
Se
O
HO
in acetone-d_6
1.32
1.31
1.24
1.23
1.16
1.00
0.98
1.21
1.15
3.08
0.98
1.05
3.05
3.49
[ppm]
[*1e9]

14

Sample Name	Pulse sequence **CARBON**	Temperature **25**	Study owner **cts**
Date collected	Solvent **cdcl3**	Spectrometer **inova500-inova500**	Operator **cts**

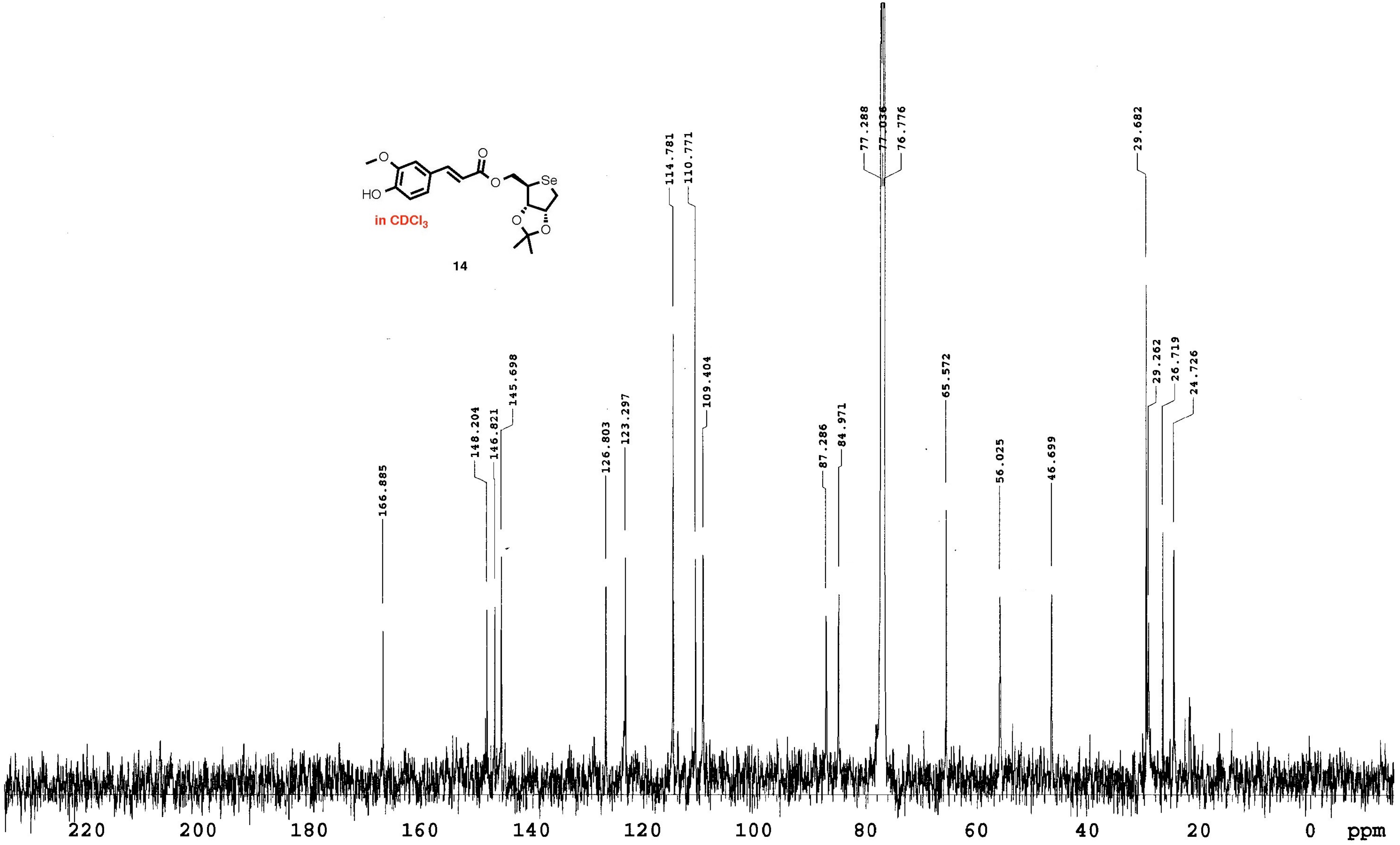

file /home/cts/vnmrsys/data/190625_AN10_RF1_20190625_01/CARBON_01

Plot date 2019-06-26

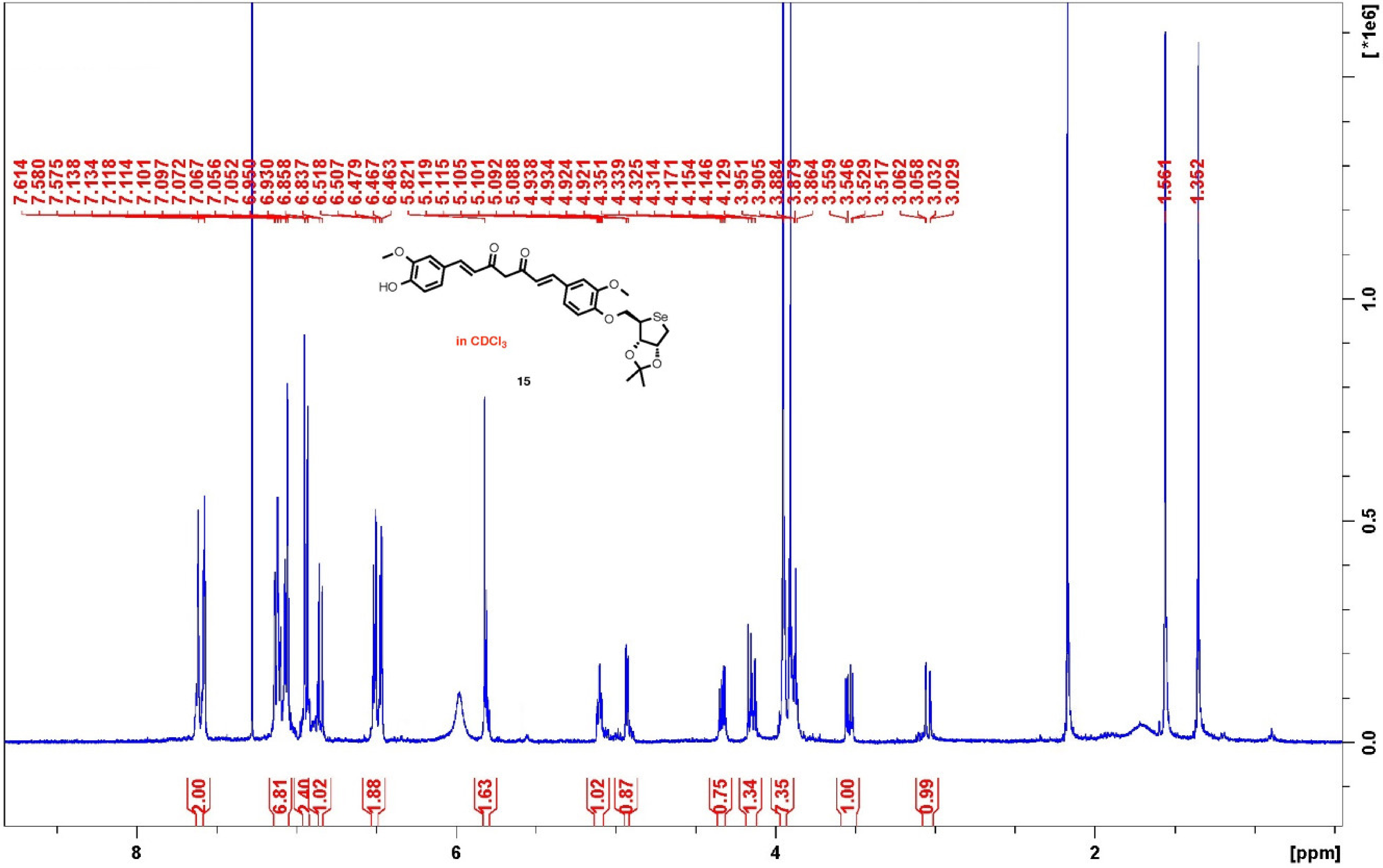
7.614
7.580
7.575
7.138
7.134
7.118
7.114
7.101
7.097
7.072
7.067
7.056
7.052
6.950
6.930
6.858
6.837
6.518
6.507
6.479
6.467
6.463
5.821
5.119
5.115
5.105
5.101
5.092
5.088
4.938
4.934
4.924
4.921
4.351
4.339
4.325
4.314
4.171
4.154
4.146
4.129
3.951
3.905
3.884
3.879
3.864
3.559
3.546
3.529
3.517
3.062
3.058
3.032
3.029
1.564
1.352
HO
O
Se
in $CDCl_3$
15
2.00
6.81
2.40
1.02
1.88
1.63
1.02
0.87
0.75
1.34
7.35
1.00
0.99
8
6
4
2
[ppm]
[*1e6]
1.0
0.5
0.0

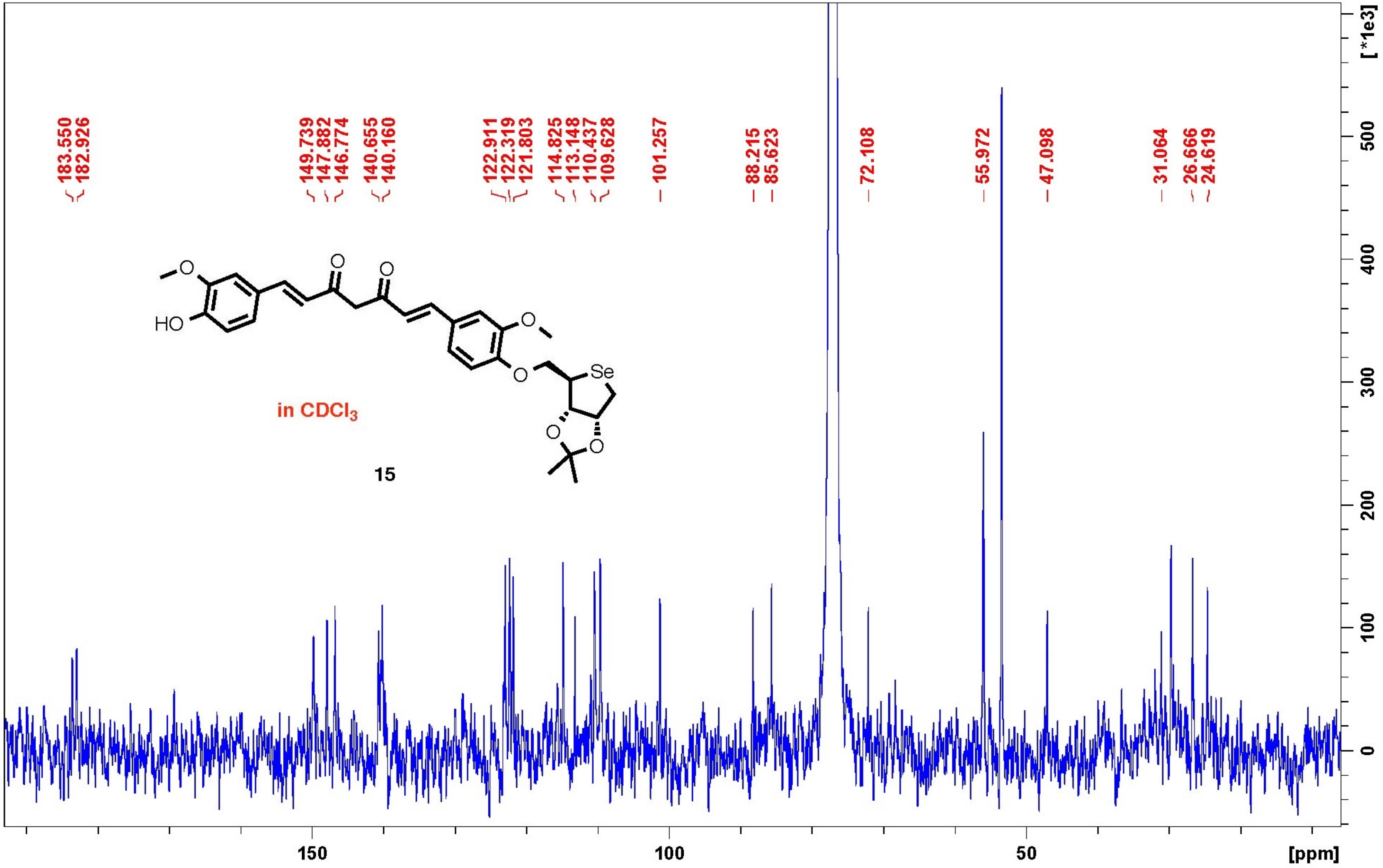
183.550
182.926
149.739
147.882
146.774
140.655
140.160
122.911
122.319
121.803
114.825
113.148
110.437
109.628
101.257
88.215
85.623
72.108
55.972
47.098
31.064
26.666
24.619
in CDCl3
15
[*1e3]
[ppm]

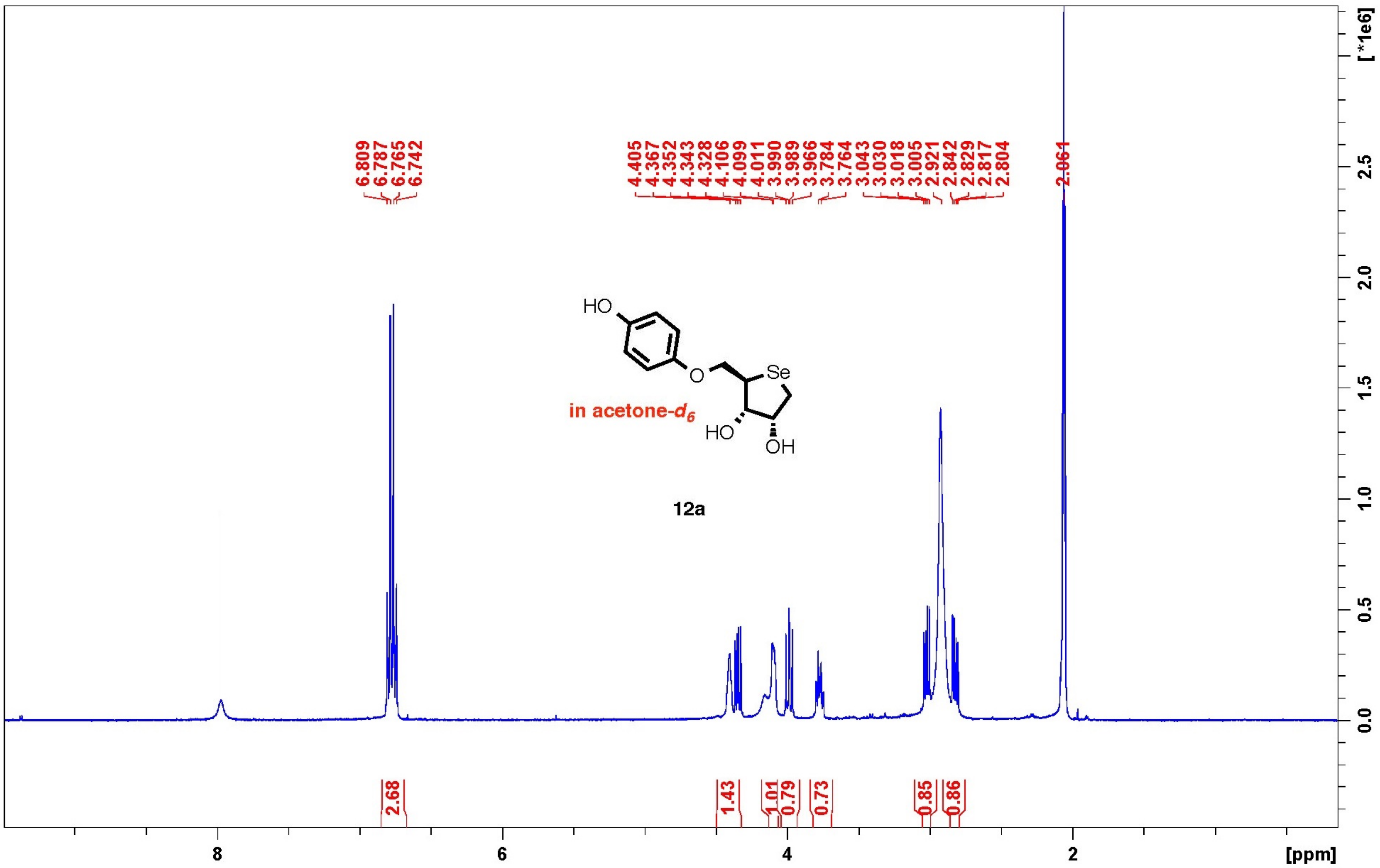

6.809
6.787
6.765
6.742
4.405
4.367
4.352
4.343
4.328
4.106
4.099
4.011
3.990
3.989
3.966
3.784
3.764
3.043
3.030
3.018
3.005
2.921
2.842
2.829
2.817
2.804
2.061
HO
O
Se
HO
OH
in acetone-d6
12a
2.68
1.43
1.01
0.79
0.73
0.85
0.86
8
6
4
2
[ppm]
[*1e6]
0.0
0.5
1.0
1.5
2.0
2.5

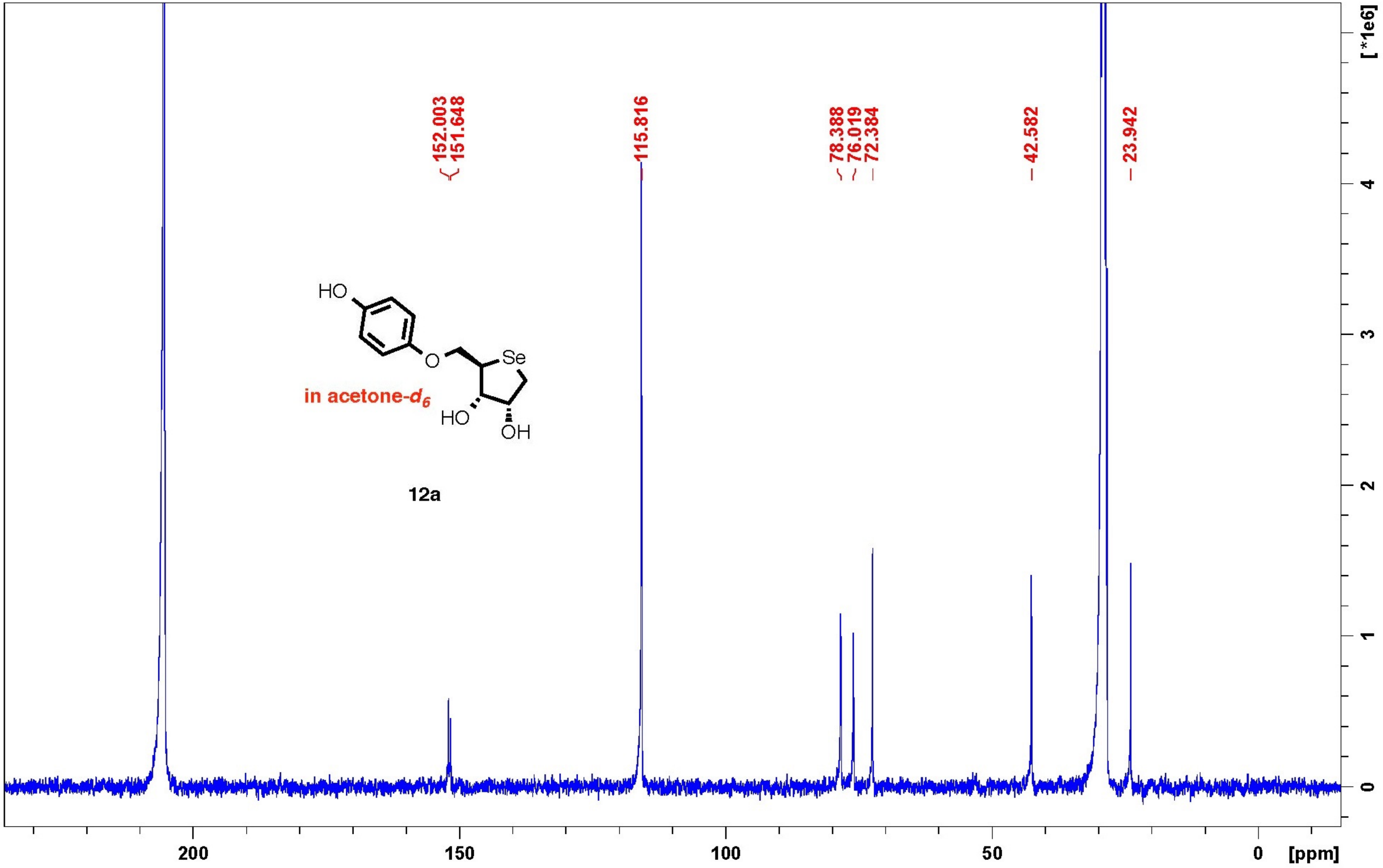
152.003
151.648
115.816
78.388
76.019
72.384
42.582
23.942
HO
O
Se
HO
OH
in acetone-d_6
12a
200
150
100
50
0
[ppm]
[*1e6]

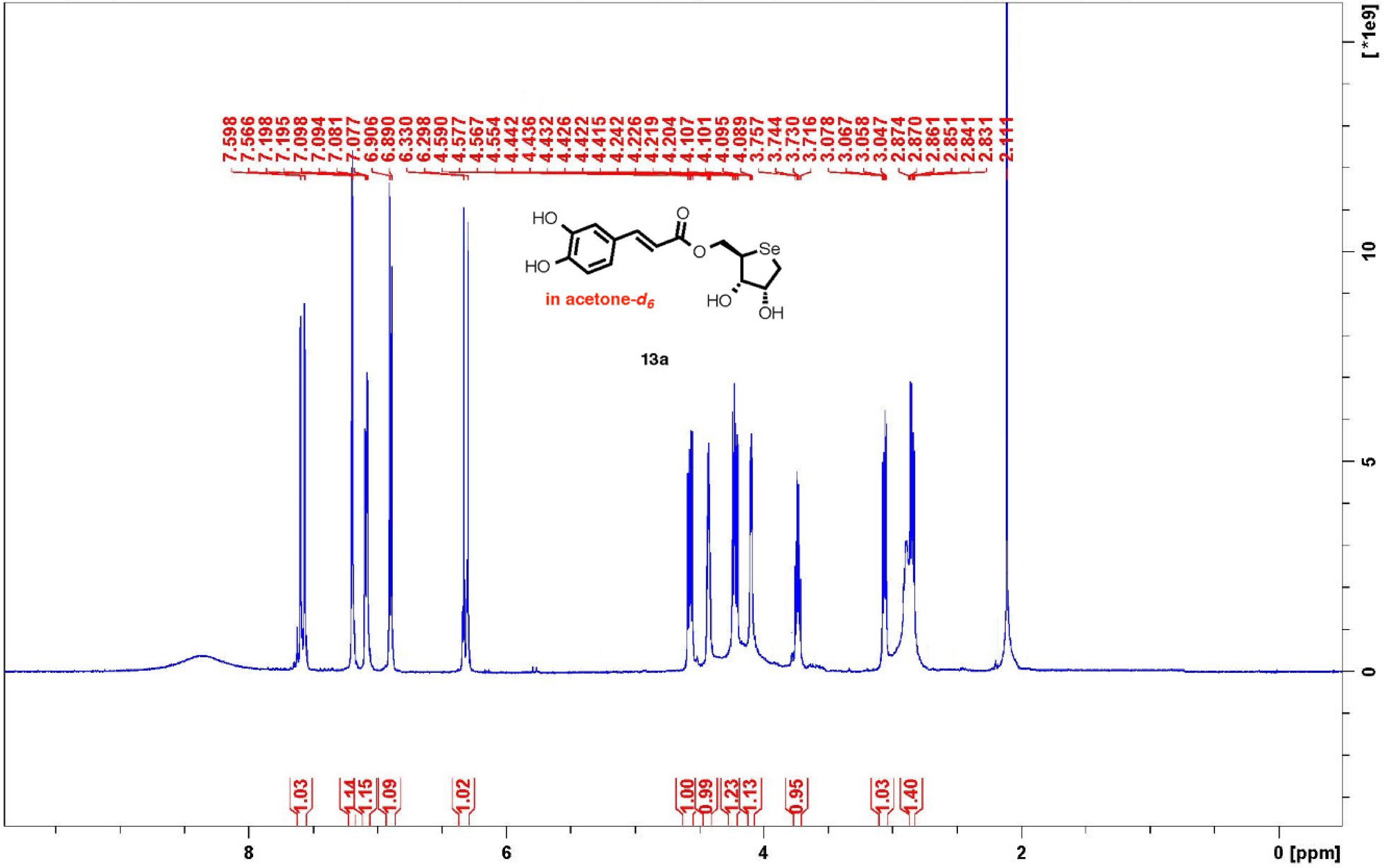

7.598
7.566
7.198
7.195
7.098
7.094
7.081
7.077
6.906
6.890
6.330
6.298
4.590
4.577
4.567
4.554
4.442
4.436
4.432
4.426
4.422
4.415
4.242
4.226
4.219
4.204
4.107
4.101
4.095
4.089
3.757
3.744
3.730
3.716
3.078
3.067
3.058
3.047
2.874
2.870
2.861
2.851
2.841
2.831
2.111
in acetone-d_6
13a
1.03
1.14
1.15
1.09
1.02
1.00
0.99
1.23
1.13
0.95
1.03
1.40
8
6
4
2
0 [ppm]
[*1e9]
10
5
0

Sample Name	Pulse sequence **CARBON**	Temperature **25**	Study owner **cts**
Date collected	Solvent **acetone**	Spectrometer **inova500-inova500**	Operator **cts**

in acetone-d_6

13a

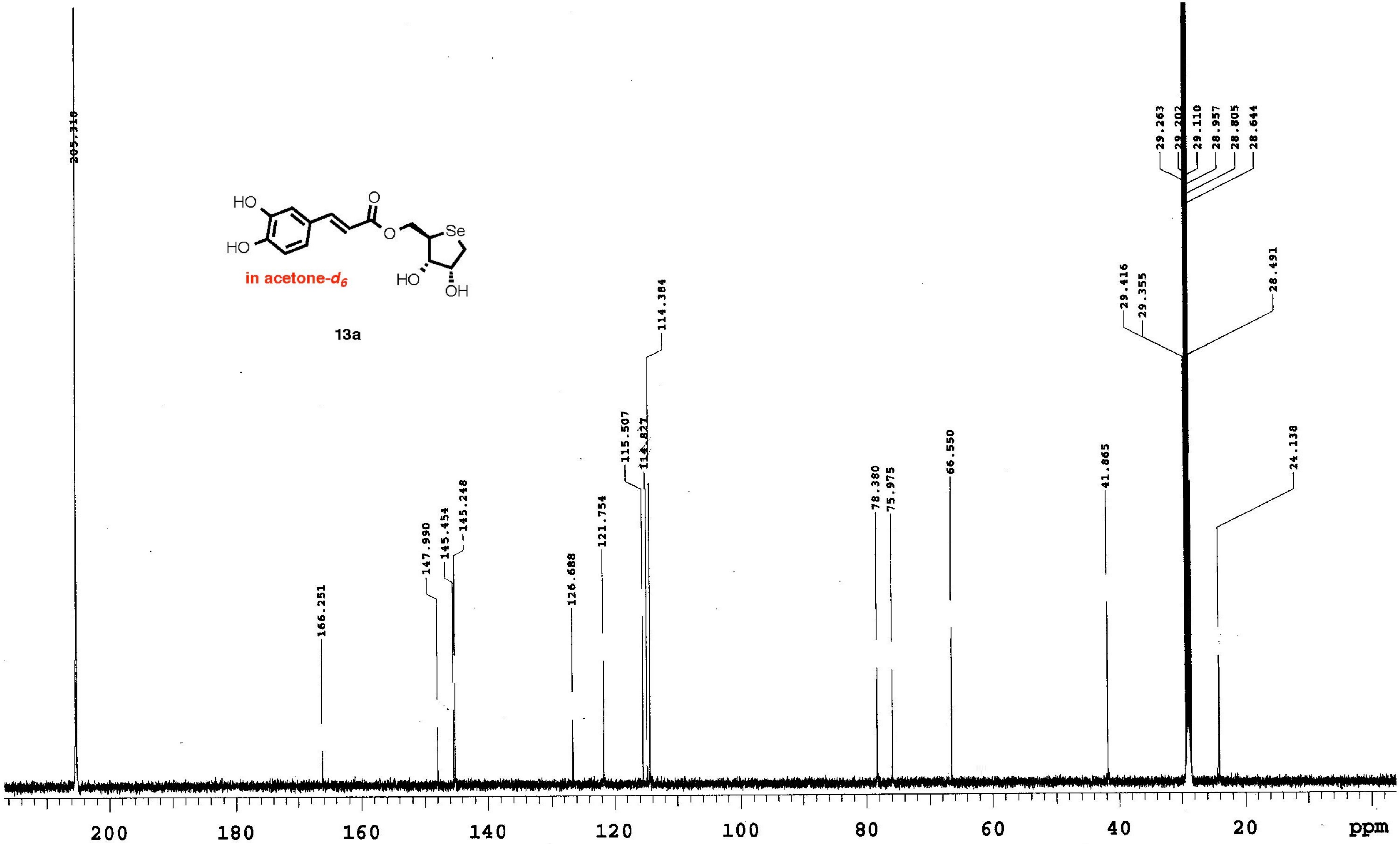

Data file /home/cts/vnmrsys/data/Selenio_20/200916_AO43_RF7_20200916_01/CARBON_01.fid

Plot date 2020-09-18

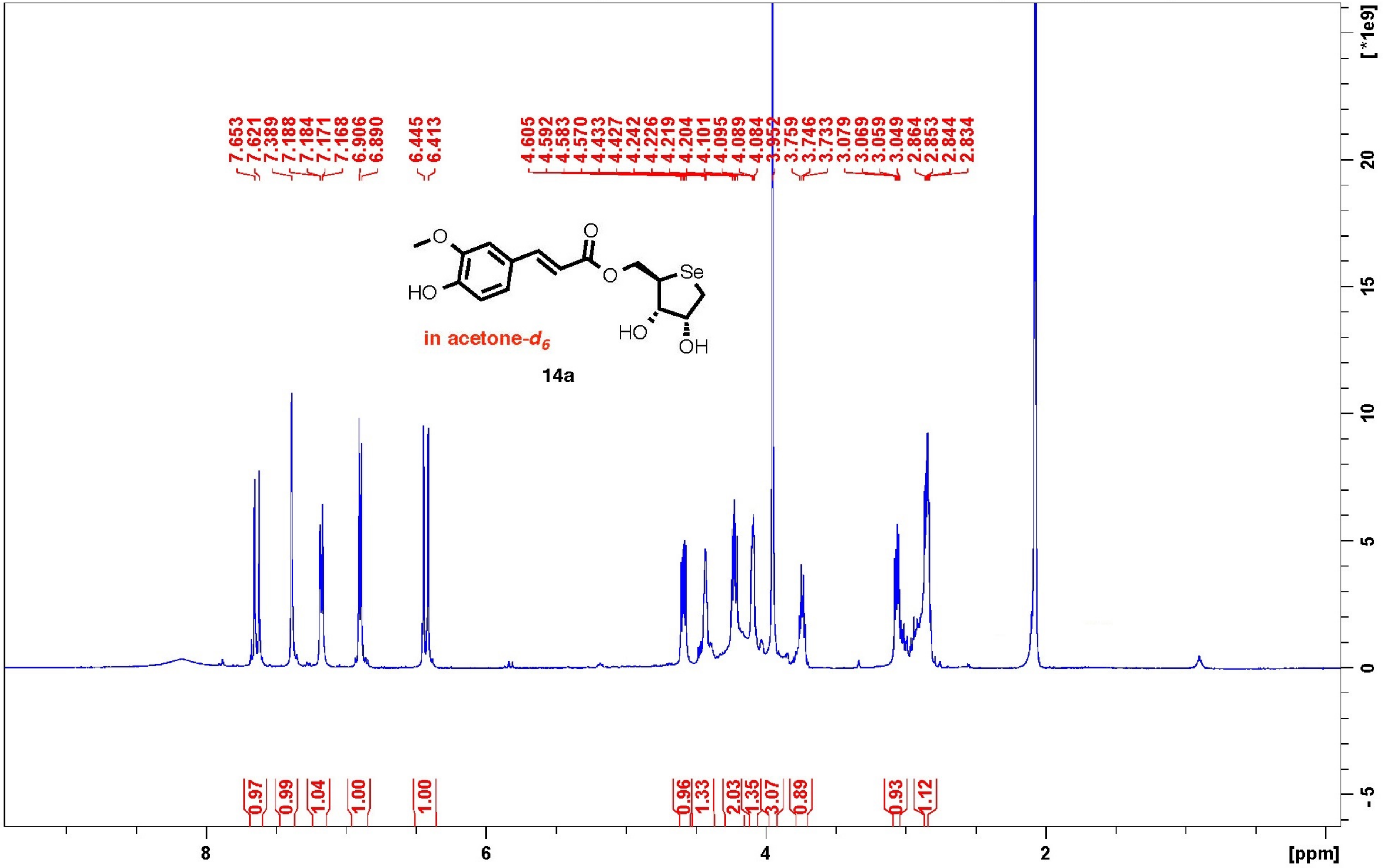
7.653
7.621
7.389
7.188
7.184
7.171
7.168
6.906
6.890
6.445
6.413
4.605
4.592
4.583
4.570
4.433
4.427
4.242
4.226
4.219
4.204
4.101
4.095
4.089
4.084
3.952
3.759
3.746
3.733
3.079
3.069
3.059
3.049
2.864
2.853
2.844
2.834
O
O
O
HO
Se
HO
OH
in acetone-d6
14a
0.97
0.99
1.04
1.00
1.00
0.96
1.33
2.03
1.35
3.07
0.89
0.93
1.12
8
6
4
2
[ppm]
[*1e9]
20
15
10
5
0
-5

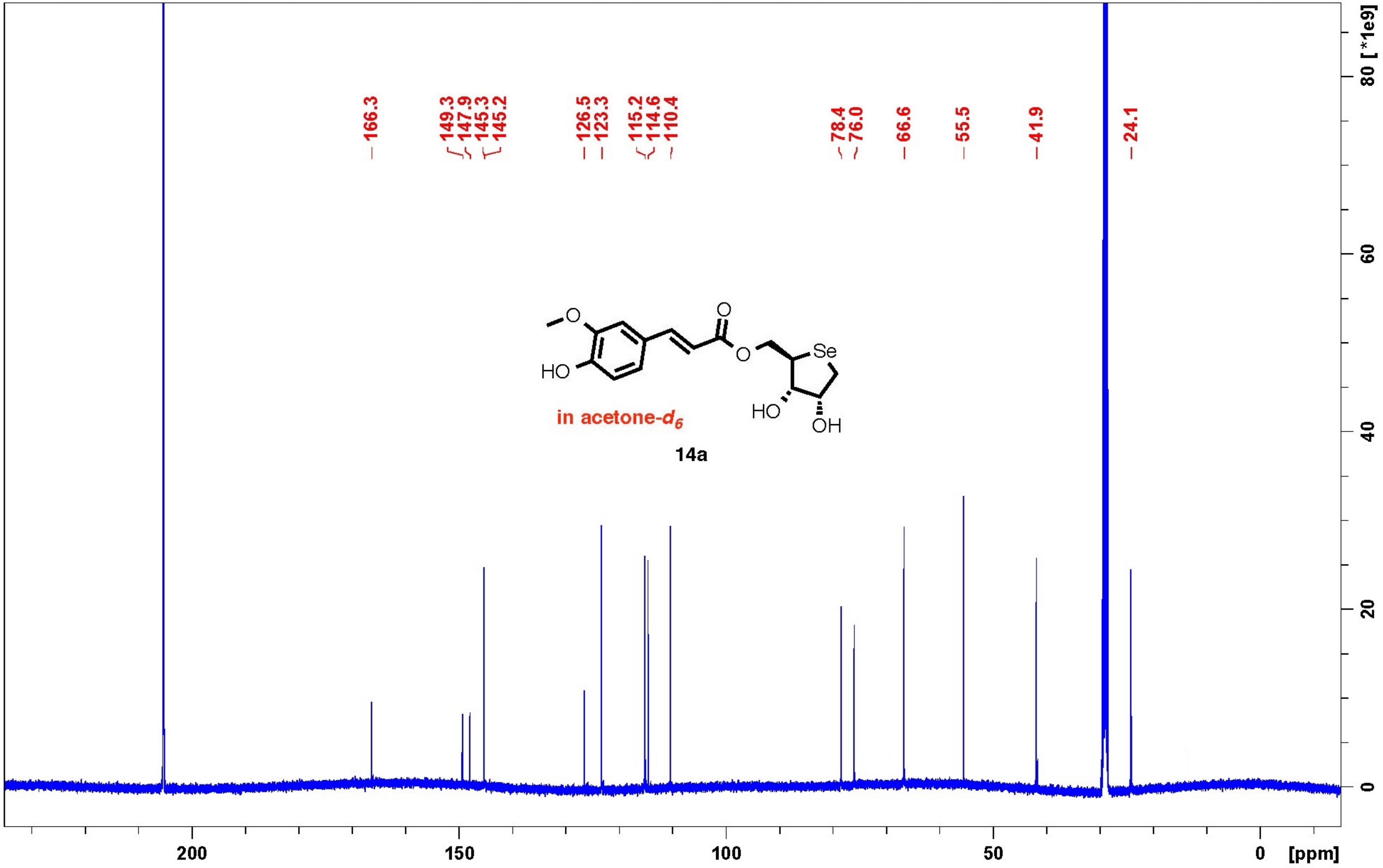

166.3
149.3
147.9
145.3
145.2
126.5
123.3
115.2
114.6
110.4
78.4
76.0
66.6
55.5
41.9
24.1
80 [*1e9]
60
40
20
0
in acetone-d_6
14a
200
150
100
50
0
[ppm]
